# Targeted deep sequencing of urothelial bladder cancers and associated urinary DNA: a 23‐gene panel with utility for non‐invasive diagnosis and risk stratification

**DOI:** 10.1111/bju.14808

**Published:** 2019-06-19

**Authors:** Douglas G. Ward, Naheema S. Gordon, Rebecca H. Boucher, Sarah J. Pirrie, Laura Baxter, Sascha Ott, Lee Silcock, Celina M. Whalley, Joanne D. Stockton, Andrew D. Beggs, Mike Griffiths, Ben Abbotts, Hanieh Ijakipour, Fathimath N. Latheef, Robert A. Robinson, Andrew J. White, Nicholas D. James, Maurice P. Zeegers, K. K. Cheng, Richard T. Bryan

**Affiliations:** ^1^ Institute of Cancer and Genomic Sciences University of Birmingham Birmingham UK; ^2^ Department of Computer Science University of Warwick Coventry UK; ^3^ Nonacus Limted Birmingham Research Park Birmingham UK; ^4^ West Midlands Regional Genetics Laboratory Birmingham Women's and Children's NHS Foundation Trust Birmingham UK; ^5^ NUTRIM School for Nutrition and Translational Research in Metabolism and CAPHRI Care and Public Health Research Institute Maastricht University Maastricht The Netherlands; ^6^ Institute of Applied Health Research University of Birmingham Birmingham UK

**Keywords:** mutations, diagnosis, prognosis, detection, urine, DNA, #BladderCancer, #blcsm

## Abstract

**Objectives:**

To develop a focused panel of somatic mutations (SMs) present in the majority of urothelial bladder cancers (UBCs), to investigate the diagnostic and prognostic utility of this panel, and to compare the identification of SMs in urinary cell‐pellet (cp)DNA and cell‐free (cf)DNA as part of the development of a non‐invasive clinical assay.

**Patients and Methods:**

A panel of SMs was validated by targeted deep‐sequencing of tumour DNA from 956 patients with UBC. In addition, amplicon and capture‐based targeted sequencing measured mutant allele frequencies (MAFs) of SMs in 314 urine cpDNAs and 153 urine cfDNAs. The association of SMs with grade, stage, and clinical outcomes was investigated by univariate and multivariate Cox models. Concordance between SMs detected in tumour tissue and cpDNA and cfDNA was assessed.

**Results:**

The panel comprised SMs in 23 genes: *TERT* (promoter), *FGFR3*,*PIK3CA*,*TP53*,*ERCC2*,*RHOB*,*ERBB2*,*HRAS*,*RXRA*,*ELF3*,*CDKN1A*,*KRAS*,*KDM6A*,*AKT1*,*FBXW7*,*ERBB3*,*SF3B1*,*CTNNB1*,*BRAF*,* C3orf70*,*CREBBP*,*CDKN2A*, and *NRAS*; 93.5–98.3% of UBCs of all grades and stages harboured ≥1 SM (mean: 2.5 SMs/tumour). *RAS* mutations were associated with better overall survival (*P* = 0.04). Mutations in *RXRA, RHOB* and *TERT* (promoter) were associated with shorter time to recurrence (*P* < 0.05). MAFs in urinary cfDNA and cpDNA were highly correlated; using a capture‐based approach, >94% of tumour SMs were detected in both cpDNA and cfDNA.

**Conclusions:**

SMs are reliably detected in urinary cpDNA and cfDNA. The technical capability to identify very low MAFs is essential to reliably detect UBC, regardless of the use of cpDNA or cfDNA. This 23‐gene panel shows promise for the non‐invasive diagnosis and risk stratification of UBC.

**Abbreviations**
AKT1: AKT serine/threonine kinase 1
BCPP: Bladder Cancer Prognosis Programme
BRAF: B‐Raf proto‐oncogene, serine/threonine kinase
C3orf70: chromosome 3 open reading frame 70
CDKN1A: cyclin‐dependent kinase inhibitor 1A
CDKN2A: cyclin‐dependent kinase inhibitor 2A
cfDNA: cell‐free DNA
CIS: carcinoma *in situ* (Tis)
cpDNA: cell‐pellet DNA
CREBBP: CREB binding protein
CTNNB1: catenin β1
EAU: European Association of Urology
ELF3: E74 like ETS transcription factor 3
EORTC: European Organisation for the Research and Treatment of Cancer
ERBB2: Erb‐B2 receptor tyrosine kinase 2
ERBB3: Erb‐B2 receptor tyrosine kinase 3
ERCC2: ERCC excision repair 2, TFIIH core complex helicase subunit
FBXW7: F‐box and WD repeat domain containing 7
FGFR3: fibroblast growth factor receptor 3
HR: hazard ratio
HRAS: HRas proto‐oncogene, GTPase
KDM6A: lysine demethylase 6A
KRAS: KRAS proto‐oncogene, GTPase
MAF: mutant allele frequency
(N)MIBC: (non‐)muscle‐invasive bladder cancer
NRAS: NRAS proto‐oncogene, GTPase
PIK3CA: phosphatidylinositol‐4,5‐bisphosphate 3‐kinase, catalytic subunit α
pT: pathological T stage
RHOB: Ras homolog family member B
RXRA: retinoid X receptor α
SF3B1: splicing factor 3b subunit 1
SM: somatic mutation
TERT: telomerase reverse transcriptase
TP53: tumour protein P53
TURBT: transurethral resection of bladder tumour
UBC: urothelial bladder cancer
UMI: unique molecular identifiers

## Introduction

Despite intensive research into biomarkers for the non‐invasive diagnosis of urothelial bladder cancer (UBC), the mainstay of detection remains flexible cystoscopy. Commercial urine tests exist; however, none have been widely accepted into routine clinical practice due to poor performance and/or poor evidence 1, 2, 3. Many tests are based on levels of proteins or RNA and, as these are not unique to UBC or causally linked to the disease, they tend to lack specificity and are often not detectably elevated in small or low‐grade tumours 4. The ideal non‐invasive test should detect all UBCs whilst not generating false‐positive results from non‐malignant urological conditions.

DNA‐based biomarkers (methylation, single nucleotide variants, and copy number variants) can be detected in urinary DNA and could be used for the non‐invasive detection and characterisation of UBC 5. Deep sequencing has enabled both the large‐scale identification of somatic mutations (SMs) in UBC 6 and the sensitive detection of SMs in urinary DNA 7, 8, 9, 10, 11. However, whole genome sequencing at sufficient depth to detect SMs at low mutant allele frequencies (MAFs) remains expensive; thus, to make a test affordable and interpretable, targeted sequencing of the minimum number of SMs that provide sufficient information is desirable. With optimisation of biomarkers and sample processing, highly sensitive and specific tests could be developed. Notwithstanding, most urine DNA‐based studies have utilised DNA extracted from the cell pellets of centrifuged urine (cpDNA) 7, 12, 13; however, several studies have reported that cell‐free DNA (cfDNA) from supernatants of centrifuged urine better represents the genomic changes in UBC 14, 15, 16.

The primary objective of the present study was to develop a focused panel of SMs present in the majority of UBCs. Our secondary objectives were to investigate the prognostic utility of this panel and to compare the identification of these SMs in urinary cpDNA and cfDNA as a stepping‐stone to the development of a non‐invasive diagnostic and prognostic clinical assay. We used a combination of publicly available data and in‐house exome sequencing to select candidate SMs for inclusion; many of the SMs are directly involved in UBC pathogenesis 6. This panel of SMs in 23 genes was validated by amplicon deep‐sequencing of primary UBCs from 956 patients. We subsequently used deep‐sequencing to identify the tumour tissue SMs in matched urine samples comprising 314 urine cpDNAs and 153 urine cfDNAs. Amplicon sequencing and a capture‐based approach were compared for SM detection in urinary DNAs.

## Patients and methods

### SM Panel Development

Using a combination of publicly available data and in‐house exome sequencing, we designed a panel to contain the most frequent SMs using the minimum amount of sequencing. Some regions/hotspots were challenging to sequence, or did not detect mutations, and were excluded. A final panel covering promoter or exonic regions in 23 genes with 61 amplicons was defined (Table S1); these genes are: telomerase reverse transcriptase (*TERT*) (promoter), fibroblast growth factor receptor 3 (*FGFR3*), phosphatidylinositol‐4,5‐bisphosphate 3‐kinase, catalytic subunit α (*PIK3CA*), tumour protein P53 (*TP53*), ERCC excision repair 2, TFIIH core complex helicase subunit (*ERCC2*), Ras homolog family member B (*RHOB*), Erb‐B2 receptor tyrosine kinase 2 (*ERBB2*), HRas proto‐oncogene, GTPase (*HRAS*), retinoid X receptor α (*RXRA*), E74 like ETS transcription factor 3 (*ELF3*), cyclin‐dependent kinase inhibitor 1A (*CDKN1A*), KRAS proto‐oncogene, GTPase (*KRAS*), lysine demethylase 6A (*KDM6A*), AKT serine/threonine kinase 1 (*AKT1*), F‐box and WD repeat domain containing 7 (*FBXW7*), *ERBB3*, splicing factor 3b subunit 1 (*SF3B1*), catenin β1 (*CTNNB1*), B‐Raf proto‐oncogene, serine/threonine kinase (*BRAF*), chromosome 3 open reading frame 70 (*C3orf70*), CREB binding protein (*CREBBP*), *CDKN2A*, and NRAS proto‐oncogene, GTPase (*NRAS*).

### Patients and Samples

Biospecimens were collected as part of the Bladder Cancer Prognosis Programme (BCPP, ethics approval 06/MRE04/65). Patients were recruited consecutively from 2005 to 2011 from 10 hospitals in the West Midlands (UK) and gave informed consent for enrolment based upon initial cystoscopic findings suggestive of primary UBC. All patients were newly diagnosed and treatment‐naïve at biospecimen collection, and were subsequently treated and monitored according to contemporary European Association of Urology (EAU) guidelines (including re‐resection where indicated) and EAU risk groups (for non‐muscle‐invasive bladder cancer [NMIBC]). Inclusion and exclusion criteria are detailed elsewhere 17. Where necessary, tumour grade and stage records were amended according to results of early re‐resection or cystectomy. We used the 1973 grade classification as it was in universal use in the UK at the time of patient recruitment, is the basis for the European Organisation for the Research and Treatment of Cancer (EORTC) and EAU NMIBC risk tables 18, and has comparable utility to the 2004/2016 classification 19. For quality assurance, 10% of diagnostic formalin‐fixed paraffin‐embedded tumour samples were retrieved from local histopathology departments and underwent expert pathological review. All included tumours were purely or predominantly TCCs.

**Figure 1:**
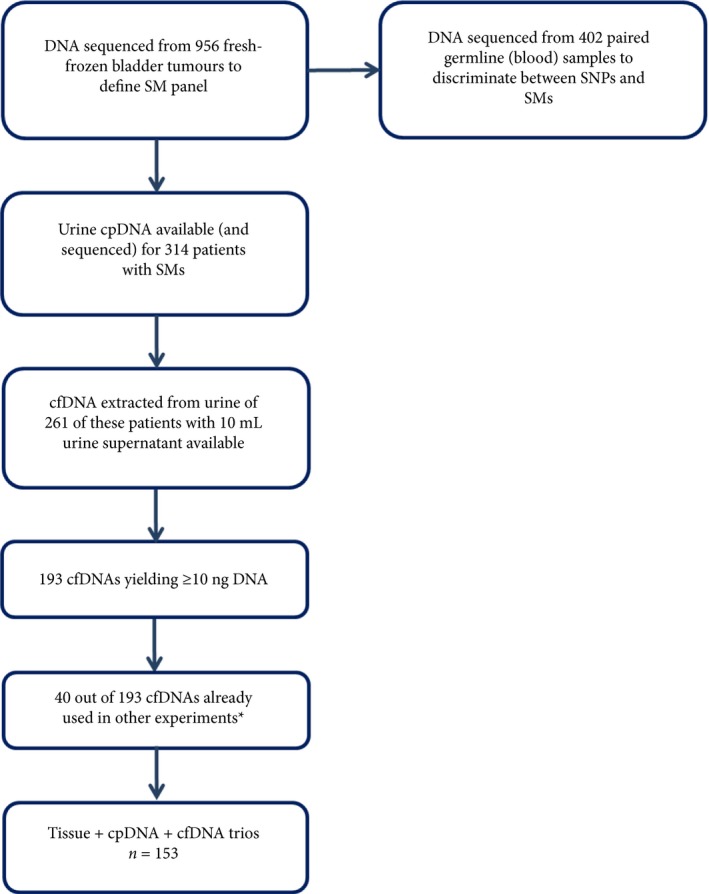
Summary of the biospecimens analysed by amplicon sequencing. SNPs, single nucleotide polymorphisms. *Used in the experiments reported by Russo et al. Bladder Cancer 2018; 4: 41–48.

Urine (30–50 mL) was placed on ice, centrifuged within 8 h (600 x *g* for 10 min), and supernatant and pellet stored at −80 °C. Tissues were collected at transurethral resection of bladder tumour (TURBT), snap‐frozen, and stored at −80 °C. DNA was extracted from tissues (25 mg) and blood (100 μL) using DNeasy Blood and Tissue kits (Qiagen, Hilden, Germany). DNA was extracted from urine pellets and supernatants (10 mL) using Quick‐DNA Urine kits (Zymo Research, Irvine, CA, USA). DNA concentrations were determined fluorimetrically (Qubit; Thermo Fisher Scientific Inc., Waltham, MA, USA). We analysed: tumour DNA from 956 patients (along with 402 matched blood samples to discriminate between mutations and polymorphisms), urine cpDNA from 314 of these 956 patients, and paired urine cfDNA from 261 of these 314 patients where >10 mL urine supernatant was available; Fig. 1.

### Library Preparation and Sequencing

Amplicon libraries were prepared by multiplex‐PCR: primers were divided between two 30‐cycle target‐specific PCRs using 5 ng DNA for each and KAPA robust polymerase. The PCR products were combined and barcoded in a 15‐cycle PCR using *Phusion* high‐fidelity polymerase 8. Up to 384 barcoded libraries were sequenced (2 × 150 bp) on a *NextSeq* mid‐output flow‐cell to a mean read depth of 5000×.

Capture‐based libraries incorporating unique molecular identifiers (UMIs) were prepared according to the manufacturer's protocol using 20 ng DNA (Cell3™ Target; Nonacus Ltd, Birmingham, UK) and sequenced as above to a mean consensus read depth of 2200×. Briefly, DNA was enzymatically fragmented, end‐repaired and A‐tailed, followed by ligation of adapters containing UMIs and incorporation of sample barcodes by PCR. Libraries were pooled and hybridised to biotinylated probes overnight, followed by bead capture, amplification, and sequencing. A detailed workflow is available at nonacus.com.

### Bioinformatics and Data Analysis

Amplicon sequencing reads were aligned to the human genome (Hg19) using *bowtie*, and reference and non‐reference read depths extracted using *bamreadcount*. Only Q >30 base‐calls were considered, and variant detection was based on the non‐reference reads >2.5% of the total read depth and a minimum of 10 non‐reference reads, as described previously 8. All mutations included in the 23‐gene panel had to meet the criteria of ≥10% MAF in ≥1 tumour and <2.5% in germline DNA. We used Sanger sequencing to confirm 50 such mutation calls, with 100% accuracy. With the exception of the well‐known *TERT* promotor mutations, only mutations classified as moderate or high impact by variant effect predictor 20 were considered. Reads from the capture‐based libraries were aligned using *Burrows‐Wheeler Alignment* tool (*BWA*), and UMI sequences were extracted as part of the i7 index read and used to annotate the aligned reads on a per original molecule basis. Using a proprietary bioinformatic pipeline, consensus reads were built where at least two reads contained the same UMI sequence and had identical genomic start and stop coordinates. Variant calls required a minimum of four supporting consensus reads.

### Prognostic Utility of Frequently Mutated Genes

Kaplan–Meier curves were constructed to investigate the effect of mutated genes on outcomes (disease‐specific survival, overall survival, and, where appropriate, progression‐free and recurrence‐free intervals). Hazard ratios (HRs) and *P* values presented with Kaplan–Meier curves were obtained by fitting univariate Cox models to the respective datasets. To account for confounding, base models including key influential factors were developed for each population and the relevant genes, then individually included in this model. If ≥2 genes were found to be significant (*P < *0.1) in a population for a specific outcome when included with the base model, further Cox models were constructed. These included every appropriate pair of genes in addition to the base model. Conditions were applied to the genes that were evaluable and to the outcomes suitable for modelling. More details are given in Appendix S1 (Supplementary data).

## Results

### Frequency of Mutations Across Stages and Grade of Disease

**Figure 2:**
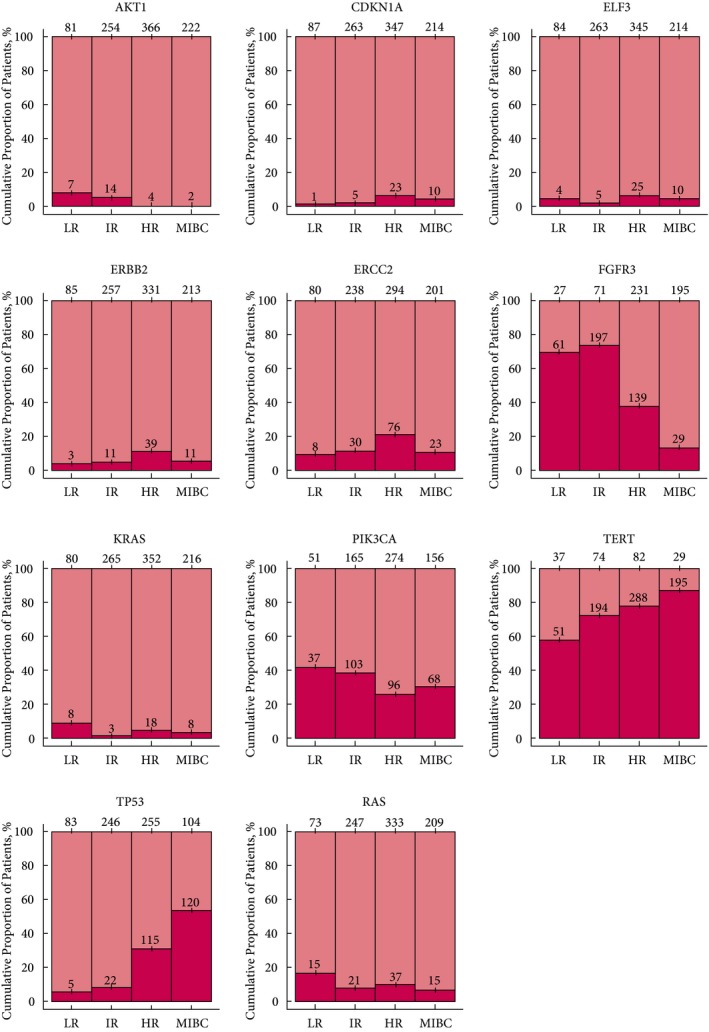
Genes in which tumour mutation frequency demonstrated significant differences between NMIBC risk groups and/or MIBC. *HRAS*,*KRAS*,*NRAS* have been combined as *RAS*. pink, wild type; magenta, mutated. LR, low‐risk NMIBC; IR, intermediate‐risk NMIBC; HR, high‐risk NMIBC.

Patient characteristics are shown in Table 1. The amplicon sequencing of hotspots/regions of 23 UBC‐associated genes in tumours from 956 patients with UBC are summarised in Table 2. A total of 916 tumours had ≥1 SM (average of 2.5 SMs/tumour), and ≥1 SM was identified in >93% of tumours of any grade or stage. We identified 451 unique SMs comprising: 384 ‘moderate impact’ variants (missense substitutions), 62 ‘high impact’ variants (likely to result in loss of functional protein), and five ‘modifier’ variants in the *TERT* promotor (Table S2). At presentation, tumours with a mutation in *FGFR3* or *AKT1* were five‐times less likely to be muscle‐invasive bladder cancer (MIBC) than tumours wild type for both genes (7% vs 38%); *TP53*‐mutated tumours were three‐times more likely to be MIBC than wild type *TP53* tumours (46% vs 15%). Mutations in 11 of the 23 genes demonstrated statistically significant differences between NMIBC risk groups and/or MIBC; these genes were *AKT1*,* CDKN1A*,* ELF3*,* ERBB2*,* ERCC2*,* FGFR3*,* KRAS*,* PIK3CA*,* TERT*,* TP53*, and *RAS* (*HRAS*,* KRAS*, and *NRAS* combined; Fig. 2).

**Table 1:** Patient and tumour characteristics.

Variable	Tumours (*n =* 956)	Urine cpDNA (*n=* 314)	Urine cfDNA (*n =* 153)	Capture‐based analyses of cpDNA and cfDNA (*n =* 45)
Age, years, median (range)	71 (26–95)	72 (26–91)	73 (26–88)	74 (44–89)
*N* (%)				
Male	748 (78.2)	247 (78.7)	126 (82.4)	32 (71.1)
Female	208 (21.8)	67 (21.3)	27 (17.6)	13 (28.9)
Grade 1 (G1)	174 (18.2)	53 (16.9)	26 (16.9)	12 (26.7)
Grade 2 (G2)	290 (30.3)	100 (31.8)	42 (27.5)	14 (31.1)
Grade 3 (G3)	478 (50.0)	153 (48.7)	80 (52.3)	19 (42.2)
Unknown (U)	14 (1.5)	8 (2.5)	5 (3.3)	0
CIS [G1/G2/G3/U]	3 [0/0/3/0] (0.3)	3 [0/0/3/0] (1.0)	3 [0/0/3/0] (2.0)	0
pTa [G1/G2/G3/U]	466 [169/224/71/2] (48.7)	143 [52/73/17/1] (45.5)	64 [25/30/9/0] (41.8)	26 [12/11/3/0] (57.8)
pT1 [G1/G2/G3/U]	263 [5/58/194/6] (27.5)	89 [1/24/60/4] (28.3)	42 [1/11/28/2] (27.5)	9 [0/2/7/0] (20.0)
≥pT2 [G1/G2/G3/U]	224 [0/9/209/6] (23.4)	79 [0/3/73/3] (25.2)	44 [0/1/40/3] (28.7)	10 [0/1/9/0] (22.2)

CIS, carcinoma *in situ*; pT, pathological T stage.

**Table 2:** Mutation frequencies across grades and stages of bladder cancer.

Gene	G1pTa(*n =* 169)	G2pTa(*n =* 224)	G3pTa (*n =* 71)	G2pT1 (*n =* 58)	G3pT1 (*n =* 194)	≥pT2(*n =* 224)	Other(*n =* 16)	Total(*n=* 956)
*TERT*	63.9	74.6	69.0	81.0	79.9	87.1	75.0	76.7
*FGFR3*	71.6	72.8	42.3	51.7	27.3	12.9	25.0	45.0
*PIK3CA*	37.9	38.8	29.6	32.8	21.6	30.4	31.3	32
*TP53*	4.7	9.4	23.9	15.5	41.8	53.6	37.5	27.4
*ERCC2*	10.1	12.9	23.9	12.1	21.1	10.3	18.8	14.3
*RHOB*	9.5	6.3	4.2	10.3	8.2	6.3	12.5	7.4
*ERBB2*	3.6	4.9	9.9	5.2	12.9	4.9	12.5	6.8
*HRAS*	7.7	4.9	1.4	3.4	6.2	2.7	0.0	4.7
*RXRA*	1.8	4.9	5.6	8.6	7.2	3.6	0.0	4.7
*ELF3*	5.3	0.4	4.2	10.3	6.7	4.5	12.5	4.6
*CDKN1A*	0.6	3.1	8.5	1.7	7.2	4.5	0.0	4.1
*KRAS*	5.3	0.9	4.2	10.3	3.6	3.6	12.5	3.9
*KDM6A*	1.2	4.9	4.2	8.6	2.1	2.2	0.0	3.1
*AKT1*	8.3	4.0	1.4	0.0	0.5	0.9	0.0	2.8
*FBXW7*	1.8	1.8	2.8	5.2	3.1	3.1	6.3	2.7
*ERBB3*	1.8	1.8	4.2	0.0	2.6	3.6	0.0	2.4
*SF3B1*	0.0	1.3	4.2	3.4	1.5	3.6	6.3	2.1
*CTNNB1*	0.0	2.7	2.8	1.7	1.0	2.7	6.3	1.9
*BRAF*	1.2	0.4	4.2	1.7	1.5	0.9	6.3	1.4
*C3orf70*	0.0	1.3	2.8	0.0	1.0	2.2	6.3	1.4
*CREBBP*	0.0	1.8	1.4	1.7	1.5	1.8	0.0	1.4
*CDKN2A*	1.2	0.0	0.0	0.0	1.5	1.8	6.3	1.0
*NRAS*	1.2	0.9	2.8	1.7	0.5	0.4	0.0	0.9
Any gene	93.5	95.5	94.4	98.3	96.9	97.8	93.8	96.0

Results are presented as the percentage of tumours in each category with a mutation in each (or any) gene. The ‘other’ category includes three cases of solitary Tis, five cases of G1pT1, and eight NMIBCs where grade was not recorded. G1, Grade 1; G2, Grade 2; G3, Grade 3.

### Prognostic Utility of Frequently Mutated Genes

**Figure 3:**
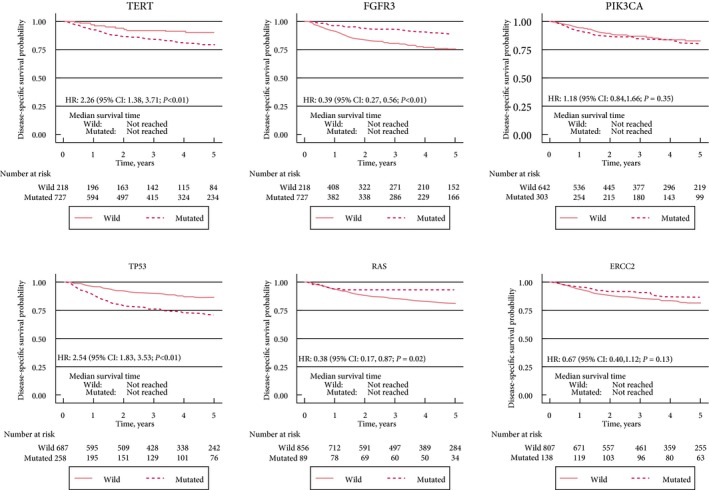
The effect of the six commonest mutated genes on disease‐specific survival across the entire cohort of 956 patients (wild = wild type).

Across the entire cohort, *TERT*,* FGFR3*,* TP53* and *RAS* were significantly associated with overall and disease‐specific survival (Fig. 3). *RAS* mutations remained significantly associated with better overall survival when adjusting for EAU risk factors (HR 0.60, 95% CI 0.37–0.97; *P = *0.04). There were insufficient events to adjust by EAU risk factors for disease‐specific survival.

**Figure 4:**
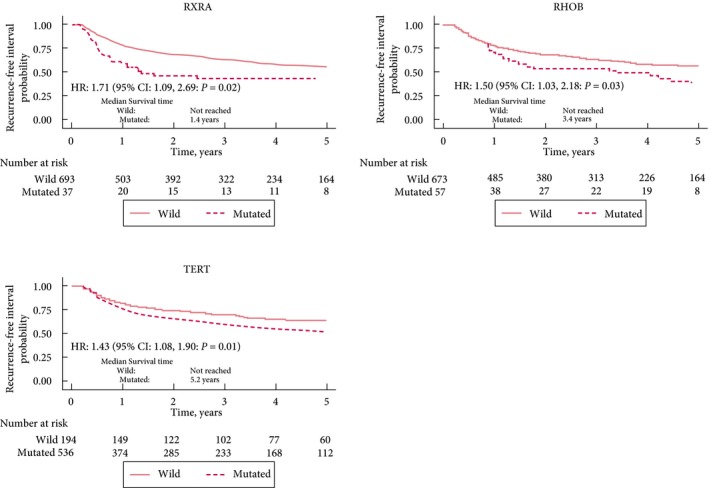
SMs with a significant influence on time to recurrence in NMIBC (wild = wild type).

The influence of mutated genes on time to recurrence and overall survival was investigated in patients with NMIBC (there were too few events to consider progression and disease‐specific survival). Mutations in *RXRA, RHOB* and the *TERT* promoter were associated with shorter time to recurrence (*P* < 0.05; Fig. 4), and remained significant after adjusting for gender and EAU risk group. *RAS* mutations were significantly associated with better overall survival after adjusting for gender and EAU risk group (*P* < 0.01).

We also analysed the influence of SMs on time to progression and disease‐specific survival in patients with high‐risk NMIBC; the association between *FGFR3* mutations and longer time to progression approached significance (HR 0.35, 95% CI 0.12–1.05; *P* = 0.06). None of the genes were significantly associated with disease‐specific survival; although survival curves for *RAS* mutant and wildtype in high‐risk NMIBC patients diverged, there were too few events to calculate statistical significance by Cox model (Appendix [Link], [Link], [Link]. Supplementary data).

In patients with MIBC, adjusted Cox models accounting for gender showed improved disease‐specific survival associated with *FGFR3* mutations (HR 1.76, 95% CI 1.05–2.93; *P* = 0.03) and worse overall survival associated with *TP53* mutations (HR 0.73, 95% CI 0.53–0.99; *P* = 0.04).

### DNA Yield from Urine Pellets and Urine Supernatants

**Figure 5:**
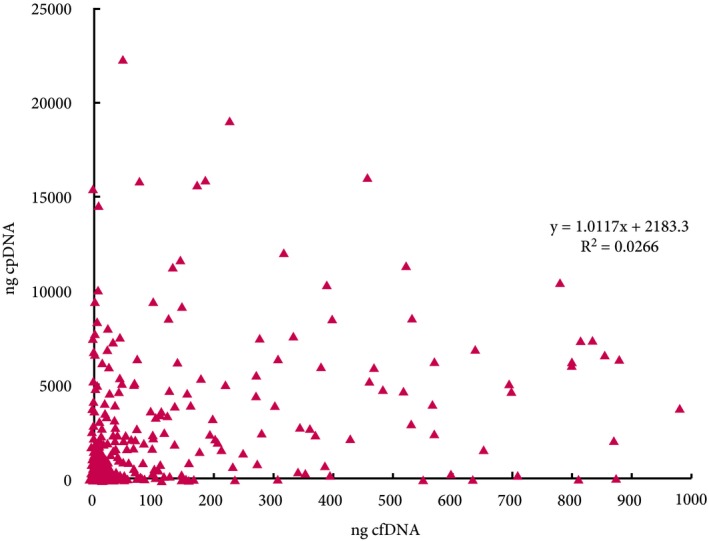
Correlation between paired urine cfDNA and cpDNA yields.

In 261 paired urinary cfDNAs and cpDNAs, the median cfDNA yield was 4.5 ng/mL of urine compared with 52 ng/mL for cpDNA. Using a minimum DNA input of 10 ng for amplicon sequencing enabled 74% of urine supernatants to be utilised, compared with >90% of pellets. Across the 261 urine samples there was no correlation between supernatant and pellet DNA yields (Fig. 5).

### Detection of SMs in Paired Tumour and cpDNA

cpDNA was available from 314 patients with SMs identified in their tumour DNA. These UBCs had 903 SMs in total with a mean of 2.9 SMs/tumour. Amplicon sequencing of cpDNAs identified 645 (71.4%) of the 903 mutations at >2.5% MAF. In all, ≥1 SM was found in 240 cpDNAs (76.4%). The median MAF across all cpDNAs was 20.6%; Grade 1, 2 and 3 disease had median MAFs of 3.7%, 22.5%, and 25.3%, respectively.

### Detection of SMs in Paired Tumour and Urine cfDNA

**Figure 6:**
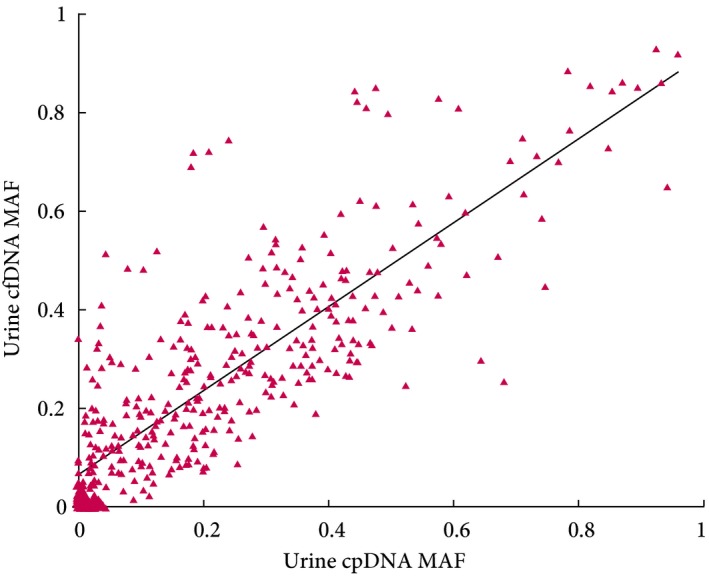
Detection of SMs in urine cfDNA and cpDNA. The graph shows the allele frequencies of mutations identified in tumours when measured in matched cfDNAs and cpDNAs by amplicon sequencing (Pearson correlation coefficient = 0.70, Spearman's rank correlation coefficient = 0.86).

**Figure 7:**
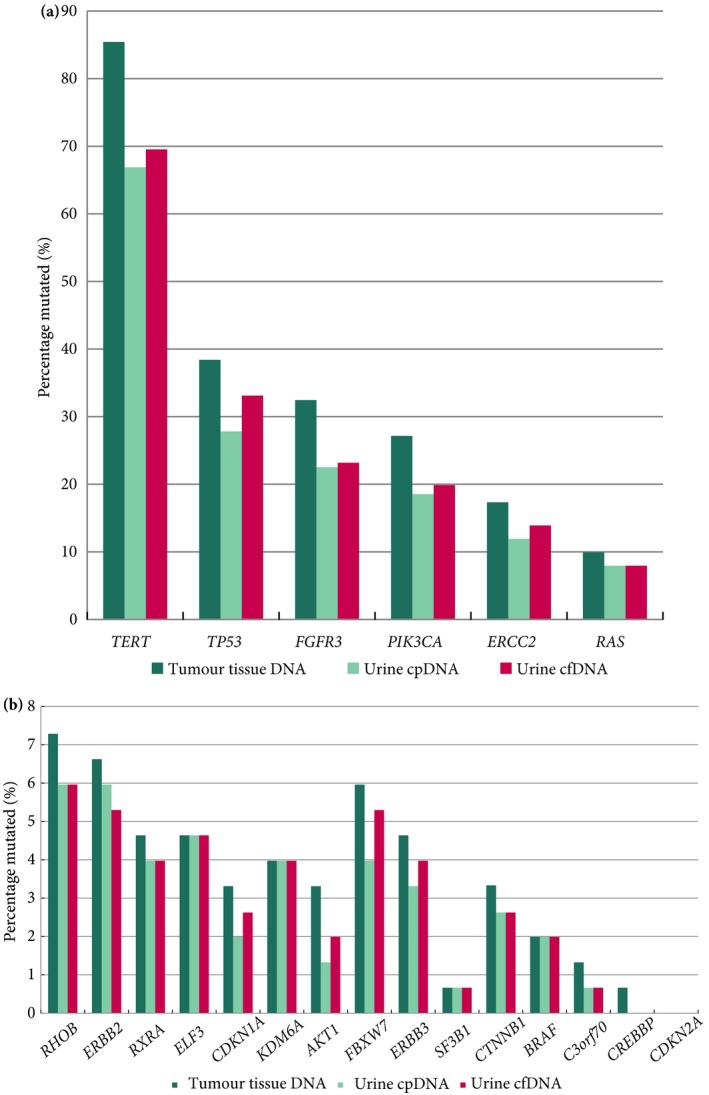
Percentage of tumours with mutations in individual genes and detection of these mutations in urinary cpDNA and cfDNA by amplicon sequencing. (a) shows the six commonest mutations (HRAS, KRAS and NRAS have been combined as RAS) and (b) shows the remaining 15. In this subset of 153 trios of tumour tissue DNA, cpDNA and cfDNA, no CDKN2A mutations were identified.

Amplicon sequencing was used to analyse cfDNA from 153 patients with tumour SMs, cpDNA data, and >10 ng cfDNA. Of 437 SMs identified in tumour DNA, 353 were detected in urinary cfDNA (80.7%), and ≥1 SM was found in 128 cfDNAs (83.8%). This compares favourably with the detection of 326 SMs in the corresponding cpDNAs (74.6%), and the detection of ≥1 SM in 118 cpDNAs (77.3%). The allele frequencies of mutations detected in 153 paired cpDNAs and cfDNAs were positively correlated (*r*_s_ = 0.86; Fig. 6), with a median MAF of 24.5% in cfDNA vs 18.9% in cpDNA (*P* < 0.001). The median MAF in Grade 1, 2 and 3 disease was 2.2%, 26.1% and 36.7% for cfDNA and 3.2%, 20.4% and 29.8% for cpDNA, respectively. The proportions of mutations identified in individual genes in each type of DNA (tumour tissue DNA, urinary cpDNA, and urinary cfDNA) are shown in Fig. 7.

### Capture‐Based cpDNA and cfDNA Analysis

**Figure 8:**
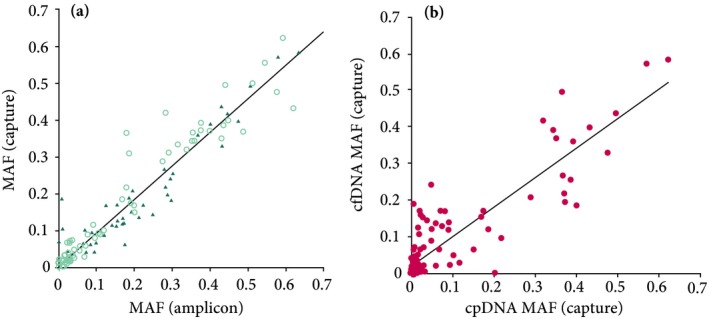
Capture‐based urine DNA analysis. (**a**) shows the correlation between MAFs in urinary DNA determined by capture‐based and amplicon‐based methods (filled triangles, cfDNA; hollow circles, cpDNA; Pearson correlation coefficient = 0.95, Spearman's rank correlation coefficient = 0.91). (**b**) shows the correlation between MAFs in cfDNA and cpDNA determined by the capture‐based method in paired samples from 45 patients (Pearson correlation coefficient = 0.79, Spearman's rank correlation coefficient = 0.76).

In paired cpDNAs and cfDNAs from 45 patients, SMs were detected by a capture‐based method (whereby consensus read building removes PCR and sequencing errors permitting detection of MAFs >10‐fold lower than standard amplicon sequencing 21). All 45 pairs of samples were from patients with SMs identified in tumour tissue; for 30 patients, SMs were not detected in cpDNA by amplicon sequencing (‘false‐negatives’) and for 15 patients they were (‘true positives’). All expected tumour SMs were detected in the true positive cpDNAs and corresponding cfDNAs; MAFs from the amplicon and capture‐based methods were closely aligned (Fig. 8a) confirming the strong correlation between cpDNA and cfDNA MAFs (Fig. 8b).

Capture‐based analysis of the ‘false negative’ cpDNAs identified SMs in 26/30 patients (86.7%), and 56/72 of all tumour SMs (77.8%); analysis of the corresponding cfDNAs identified SMs in 24/30 patients (80.0%) and 54/72 of all tumour SMs (75.0%).

## Discussion

DNA‐based urinary biomarkers have emerged as the frontrunners for the non‐invasive detection of UBC. The ideal DNA‐based non‐invasive diagnostic test for bladder cancer would utilise the minimal amount of sequencing to obtain optimal sensitivity across all grades and stages of disease, whilst utilising a DNA substrate abundant in the majority of urine samples. In the present study, we describe such a test, identifying 451 SMs in 23 genes that, overall, were present in 96% of UBCs. Many commonly mutated large tumour suppressor genes (e.g. *KDM6A*,* KMT2D*) with SMs widely distributed across the gene were unsuitable for inclusion. Notwithstanding, this panel demonstrates potential for non‐invasive detection of UBC via urinary DNA.

The distribution of common SMs across stages and grades of UBC in this cohort is consistent with previous data 22, 23. Also consistent with the literature 24, 25, 26, we found that *TP53*,* FGFR3* and *TERT* promoter mutations are predictive of survival in univariate analyses, but are not significant in multivariate analyses adjusting for accepted risk factors 1. *RAS* mutations are associated with improved survival and remain so after adjusting for EAU risk factors. As *RAS* mutations are known activators of a known oncogene, it is unlikely that they are beneficial *per se* and more likely that they co‐occur with favourable events or are mutually exclusive with unfavourable events. We caution that *RAS* mutations have not been reported as prognostic in UBC in other large datasets 6, 23. There have been contradictory reports as to whether *PIK3CA* mutations are prognostic 23, 27, 28, but we demonstrate no relationship. Additionally, we find that *RXRA, RHOB* and *TERT* mutations are all associated with decreased recurrence‐free interval in NMIBC.

We have shown that 71% of SMs harboured by UBCs can be detected in corresponding urine cpDNA by amplicon sequencing (2.5% MAF threshold) resulting in the detection of 76% of mutation‐positive tumours. Capture‐based analysis of cpDNAs confirmed that the SMs detected at >2.5% MAF by amplicon sequencing were genuine, and that decreasing the limit of detection to 0.2% MAF increases the number of SMs detected. If we had applied the capture‐based approach to all cpDNAs we hypothesise that up to 94% of all SMs could have been detected, potentially identifying 95% of mutation‐positive tumours.

Using amplicon sequencing, tumour SMs were detected in 78% of cfDNAs, and cfDNA and cpDNA MAFs were correlated, as previously demonstrated 29. There was a small (5%) but significant (*P* < 0.001) increase in average MAF in cfDNA relative to cpDNA. We verified these data using a capture‐based approach with improved analytical sensitivity; this method is less error‐prone, extremely sensitive (due to UMIs and consensus reads), and quantitative (sequencing reads can be mapped back to individual DNA molecules). Using this method we found that all tumour SMs that can be detected at >2.5% MAF in cpDNA were also detected in cfDNA, and that 80% of the SMs missed in cpDNA at >2.5% MAF can be detected in cfDNA at >0.2% MAF. We hypothesise that if the capture‐based method had been applied to all cfDNAs then up to 95% of all SMs could have been detected, potentially identifying 97% of mutation‐positive tumours.

Our present data also show that sequencing selected regions of 10s of genes (rather than 100s of genes) could provide the basis for a non‐invasive diagnostic test for UBC, with high sensitivity for all grades and stages of disease. The majority of false negative urine samples were due to undetectably low MAFs in cpDNA and cfDNA, and not due to the absence of mutations in the tumour. Thus, the technical ability to identify very low MAFs should be a key component of any such test.

Other workers have also utilised cpDNA and targeted deep sequencing for the identification of genomic alterations in urine samples from patients with UBC 7, 9, 30; however, few studies have directly compared cpDNA and cfDNA by targeted deep sequencing in this setting 11, 31. Although cpDNA is conventionally utilised for urinary biomarker studies (principally due to higher yields than cfDNA), we have shown that SM detection in urinary cfDNA works as well as (or marginally better than) SM detection in cpDNA. Notwithstanding, >25% of urine supernatants yielded <1 ng/mL DNA and were unsuitable for analysis; thus, the abundance of urinary cpDNA likely outweighs the marginal advantages of cfDNA. Preparing cpDNA and cfDNA in parallel, and then either analysing both, or cpDNA whenever possible and cfDNA in cases where cpDNA extraction fails, would reduce the number of untestable samples. To improve cfDNA yields per urine sample in the future, the development of economical and efficient methods to extract cfDNA from larger urine volumes (>100 mL) would facilitate the widespread applicability of urinary cfDNA analysis.

Our primary objective was to develop a focused panel of SMs present in the majority of UBCs and, secondarily, to investigate its prognostic utility and detection in cpDNA and cfDNA as a stepping‐stone to the development of a clinical diagnostic assay. Validation in another cohort of patients with UBC will be required to translate these findings, as well as the presentation of sensitivities and specificities from participants with and without UBC; this work is ongoing. However, we consider the data presented here to be of interest to both the UBC and liquid biopsy research communities, with additional novel findings relating to prognosis. Furthermore, recent evidence also suggests that mutations in four of the genes within our panel (*ERCC2*,* FGFR3*,* PIK3CA* and *ERBB2*) are associated with response to cisplatin‐based neoadjuvant chemotherapy for MIBC 32, 33, and FGFR inhibitors are in clinical trials for patients with advanced MIBC 34, thus demonstrating additional potential utility of our panel. However, with regard to treatment selection for FGFR inhibition (and of also relevance to ERBB2), it was noticeable that the identification of actionable mutations by amplicon sequencing was superior in tumour tissue DNA than in urinary DNA (Fig. 7); notwithstanding, the collection, shipping, handling and processing of liquid biopsies for such assays is generally easier than for conventional tumour biopsies, with the added benefits of abundance and the potential for repeat testing.

It should also be noted that patients in the present study all had primary UBC with urine samples collected pre‐TURBT and tumour samples collected at TURBT; confirmation is required regarding the sensitivities and specificities of mutation detection in UBC surveillance urine samples (both NMIBC surveillance, and MIBC surveillance following bladder‐preservation), and the potential confounding effects of urothelial field change, radiotherapy, and other urological conditions. Again, this work is ongoing.

## Conclusions

We have described key components of a potential non‐invasive diagnostic test for bladder cancer based upon a 23‐gene panel, and which also demonstrates additional utility for risk stratification and the possibility of therapeutic response prediction in specific settings. SMs can be reliably detected in urinary cpDNA and cfDNA, although the technical capability to identify very low MAFs is essential to reliably detect UBC regardless of the use of cpDNA or cfDNA. Given the higher yields of cpDNA per urine sample, cfDNA could be used to corroborate cpDNA results or if cpDNA yields are insufficient.

## Conflict of interest

R.T. Bryan has contributed to advisory boards for Olympus Medical Systems and Janssen. N.D. James has contributed to advisory boards for Merck USA and Pierre Fabre. L. Silcock is an employee of Nonacus Limited. All other authors have no conflicts to declare.

## Supporting information


**Table S1.** Final panel covering promoter or exonic regions in 23 genes with 61 amplicons.

**Table S2.** Mutation types and sites in bladder cancer‐associated genes. The table shows the number of moderate impact (missense mutations) and high impact mutations (start/stop gain/loss and splice site mutations) and the amino acid positions of hotspots.

**Appendix S1.** Supplementary data.

**Appendix S2.** REMARK criteria.

Supplementary Material
