# Supplementary material for: Targeted deep sequencing of urothelial bladder cancers and associated urinary DNA: a 23‐gene panel with utility for non‐invasive diagnosis and risk stratification
Source: BJU Int. 2019 Jun 19;124(3):532–44. doi: 10.1111/bju.14808 (PMC6772022; doi:10.1111/bju.14808)
Supplement: Supplementary file 1 — Table S1. Final panel covering promoter or exonic regions in 23 genes with 61 amplicons. [file BJU-124-532-s001.docx]

| forward primer | reverse primer | Amplicon coordinates | Sequenced bases | GENE | Amino acids covered |
| --- | --- | --- | --- | --- | --- |
| CGCCTGTCCTCATGTATTGGT | TCCACACCCCCAGGATTCTTA | [chr1:115256485+115256612](http://rohsdb.cmb.usc.edu/GBshape/cgi-bin/hgTracks?hgsid=1297593_O3lTZUTaPCQnL67b2i3SXH5i64QU&db=hg19&position=chr1:115256485-115256612&hgPcrResult=pack) | chr1:115256506-115256591 | NRAS | arg41-arg68 |
| CACTGGGCCTCACCTCTATG | GGCTCGCCAATTAACCCTGA | [chr1:115258658+115258835](http://rohsdb.cmb.usc.edu/GBshape/cgi-bin/hgTracks?hgsid=1297593_O3lTZUTaPCQnL67b2i3SXH5i64QU&db=hg19&position=chr1:115258658-115258835&hgPcrResult=pack) | chr1:115258678-115258815 | NRAS | met1-pro34 |
| CCTGAGCTGAGCCTGTTTCCC | ACATCGTGAGAAGTCAATGGCG | [chr1:201981048+201981206](https://genome.ucsc.edu/cgi-bin/hgTracks?hgsid=609383779_qtc7RsLFCqQRDVnLP1pWCYj0zcL0&db=hg19&position=chr1:201981048-201981206&hgPcrResult=pack) | chr1:201981069-201981184 | ELF3 | glu55-ala87 |
| CTTGAGGGAGGGATTAGGGGA | GGGAAGGAAGGAGAAAACGGG | [chr1:201981423-201981592](https://genome.ucsc.edu/cgi-bin/hgTracks?hgsid=540294889_SLBr2JfGF7eJxgDpd7hG5PC9jM0J&db=hg19&position=chr1:201981423-201981592&hgPcrResult=pack) | chr1:201981444-201981571 | ELF3 | thr29-phe159 |
| CTGAGTTCTCACCTCCTCTTCCC | CTGCGGTGGAGACGTCAGAG | [chr1:201981743+201981887](http://rohsdb.cmb.usc.edu/GBshape/cgi-bin/hgTracks?hgsid=1298971_4JiBau2sRe8Wp3J3JR0B2JKAtXNp&db=hg19&position=chr1:201981743-201981887&hgPcrResult=pack) | chr1:201981766-201981867 | ELF3 | asp160-gly192 |
| GGGACACCTGGATGGCAAA | CGGAGCGCAGGAACTTGAAGA | [chr1:201982916+201983076](http://rohsdb.cmb.usc.edu/GBshape/cgi-bin/hgTracks?hgsid=1298971_4JiBau2sRe8Wp3J3JR0B2JKAtXNp&db=hg19&position=chr1:201982916-201983076&hgPcrResult=pack) | chr1:201982935-201983055 | ELF3 | ala269-gly301 |
| ACGAGGGCCTCATGAAGTGG | TCCGGCTGTATCGTGAGGGT | [chr1:201983017-201983189](https://genome.ucsc.edu/cgi-bin/hgTracks?hgsid=540294889_SLBr2JfGF7eJxgDpd7hG5PC9jM0J&db=hg19&position=chr1:201983017-201983189&hgPcrResult=pack) | chr1:201983037-201983169 | ELF3 | glu296-arg334 |
| CCTCTGACCATCCTTCTCTTCA | AGTTTGGTCCCGGGTATAGT | [chr1:201984310-201984479](https://genome.ucsc.edu/cgi-bin/hgTracks?hgsid=554786469_EaV9PnD7xSCdiWpGCnDNABpmluu8&db=hg19&position=chr1:201984310-201984479&hgPcrResult=pack) | chr1:201984332-201984459 | ELF3 | tyr355-*372 |
| AACACACACAGGAAGCCCTC | CCTGAGCCCTGTCCTCCT | [chr11:533811+533966](http://rohsdb.cmb.usc.edu/GBshape/cgi-bin/hgTracks?hgsid=1297593_O3lTZUTaPCQnL67b2i3SXH5i64QU&db=hg19&position=chr11:533811-533966&hgPcrResult=pack) | chr11:533831-533948 | HRAS | asp38-gly75 |
| GGGGTCGTATTCGTCCACAA | GAGACCCTGTAGGAGGACCC | [chr11:534221+534366](http://rohsdb.cmb.usc.edu/GBshape/cgi-bin/hgTracks?hgsid=1297593_O3lTZUTaPCQnL67b2i3SXH5i64QU&db=hg19&position=chr11:534221-534366&hgPcrResult=pack) | chr11:534241-534346 | HRAS | met1-his27 |
| CAAAGAAAGCCCTCCCCAGT | TGTGTTTCTCCCTTCTCAGGATT | [chr12:25380219+25380365](http://rohsdb.cmb.usc.edu/GBshape/cgi-bin/hgTracks?hgsid=1297593_O3lTZUTaPCQnL67b2i3SXH5i64QU&db=hg19&position=chr12:25380219-25380365&hgPcrResult=pack) | chr12:25380239-25380342 | KRAS | tyr40-arg73 |
| TGTTGGATCATATTCGTCCACAA | TAAGGCCTGCTGAAAATGACTG | [chr12:25398214+25398333](http://rohsdb.cmb.usc.edu/GBshape/cgi-bin/hgTracks?hgsid=1297593_O3lTZUTaPCQnL67b2i3SXH5i64QU&db=hg19&position=chr12:25398214-25398333&hgPcrResult=pack) | chr12:25398237-25398311 | KRAS | tyr4-his27 |
| TCTGCTCTGTCACAGTGGATT | CGTGGCTGGAGTTGGTGTTA | [chr12:56478764+56478932](http://rohsdb.cmb.usc.edu/GBshape/cgi-bin/hgTracks?hgsid=1297593_O3lTZUTaPCQnL67b2i3SXH5i64QU&db=hg19&position=chr12:56478764-56478932&hgPcrResult=pack) | chr12:56478785-56478912 | ERBB3 | arg81-asn122 |
| TCCAAACTCACCCTAATTTCTTCAC | ACCCTGAGTTAAACATGTGCCT | [chr16:3788549+3788706](http://rohsdb.cmb.usc.edu/GBshape/cgi-bin/hgTracks?hgsid=1297593_O3lTZUTaPCQnL67b2i3SXH5i64QU&db=hg19&position=chr16:3788549-3788706&hgPcrResult=pack) | chr16:3788574-3788684 | CREBBP | arg1428-tyr1460 |
| GGCACGGTAATGCTGCTCAT | GCTTGCTGCACTTCTCACAC | [chr17:37868109-37868288](https://genome.ucsc.edu/cgi-bin/hgTracks?hgsid=572275029_HpWO3bauFUo9oU1pgDTcfHqcwQ4t&db=hg19&position=chr17:37868109-37868288&hgPcrResult=pack) | chr17:37868129-37868268 | ERBB2 | asn302-gln329 |
| CCCCAAACTAGCCCTCAATCC | AGCAGTCTCCGCATCGTGTA | [chr17:37879528-37879697](https://genome.ucsc.edu/cgi-bin/hgTracks?hgsid=541941303_qi2VcduKnuPYlzyg4yp7qvcNHQax&db=hg19&position=chr17:37879528-37879697&hgPcrResult=pack) | chr17:37879549-37879677 | ERBB2 | pro650-lys684 |
| GCACTGACCCACCACCC | ACCTGGCCCTGACCTTGTA | [chr17:37879763-37879926](https://genome.ucsc.edu/cgi-bin/hgTracks?hgsid=541941303_qi2VcduKnuPYlzyg4yp7qvcNHQax&db=hg19&position=chr17:37879763-37879926&hgPcrResult=pack) | chr17:37879780-37879907 | ERBB2 | leu696-val734 |
| ACTCATATCCTCCTCTTTCTGCC | CATGGGGTCCTTCCTGTCCTC | [chr17:37880139+37880311](http://rohsdb.cmb.usc.edu/GBshape/cgi-bin/hgTracks?hgsid=1297053_YhBDQLfckaSyNagANWNuzEyjhKvW&db=hg19&position=chr17:37880139-37880311&hgPcrResult=pack) | chr17:37880162-37880290 | ERBB2 | gly737-asp769 |
| TACATGGGTGCTTCCCATTCC | CCCCATCTGCATGGTACTCT | [chr17:37881279-37881452](https://genome.ucsc.edu/cgi-bin/hgTracks?hgsid=541941303_qi2VcduKnuPYlzyg4yp7qvcNHQax&db=hg19&position=chr17:37881279-37881452&hgPcrResult=pack) | chr17:37881300-37881432 | ERBB2 | gly832-glu874 |
| TCACTCACCTGGAGTGAGCC | ATATACTTACTTCTCCCCCTCCTCT | [chr17:7573919+7574068](http://rohsdb.cmb.usc.edu/GBshape/cgi-bin/hgTracks?hgsid=1298971_4JiBau2sRe8Wp3J3JR0B2JKAtXNp&db=hg19&position=chr17:7573919-7574068&hgPcrResult=pack) | chr17:7573939-7574043 | TP53 | ile332-ser362 |
| TCCACTTGATAAGAGGTCCCA | TGCAGTTATGCCTCAGATTCAC | [chr17:7576821+7576978](http://rohsdb.cmb.usc.edu/GBshape/cgi-bin/hgTracks?hgsid=1298971_4JiBau2sRe8Wp3J3JR0B2JKAtXNp&db=hg19&position=chr17:7576821-7576978&hgPcrResult=pack) | chr17:7576842-7576956 | TP53 | ala307-gln331 |
| TCCTCCACCGCTTCTTGTCC | TTGAGGTGCGTGTTTGTGCC | [chr17:7576988+7577129](http://rohsdb.cmb.usc.edu/GBshape/cgi-bin/hgTracks?hgsid=1298971_4JiBau2sRe8Wp3J3JR0B2JKAtXNp&db=hg19&position=chr17:7576988-7577129&hgPcrResult=pack) | chr17:7577008-7577109 | TP53 | cys277-arg306 |
| GGTGAGGCTCCCCTTTCTTG | TCCTTACTGCCTCTTGCTTCTC | [chr17:7577049+7577195](http://rohsdb.cmb.usc.edu/GBshape/cgi-bin/hgTracks?hgsid=1298971_4JiBau2sRe8Wp3J3JR0B2JKAtXNp&db=hg19&position=chr17:7577049-7577195&hgPcrResult=pack) | chr17:7577069-7577173 | TP53 | gly262-leu289 |
| AGTGTGCAGGGTGGCAAG | CCTCATCTTGGGCCTGTGTT | [chr17:7577470+7577637](http://rohsdb.cmb.usc.edu/GBshape/cgi-bin/hgTracks?hgsid=1298971_4JiBau2sRe8Wp3J3JR0B2JKAtXNp&db=hg19&position=chr17:7577470-7577637&hgPcrResult=pack) | chr17:7577488-7577617 | TP53 | val225-ser260 |
| GAGACCCCAGTTGCAAACC | GCCTCTGATTCCTCACTGATTG | [chr17:7578154+7578318](http://rohsdb.cmb.usc.edu/GBshape/cgi-bin/hgTracks?hgsid=1298971_4JiBau2sRe8Wp3J3JR0B2JKAtXNp&db=hg19&position=chr17:7578154-7578318&hgPcrResult=pack) | chr17:7578173-7578296 | TP53 | leu188-glu224 |
| AGCAATCAGTGAGGAATCAGAGG | CCATGGCCATCTACAAGCAGT | [chr17:7578295+7578454](http://rohsdb.cmb.usc.edu/GBshape/cgi-bin/hgTracks?hgsid=1298971_4JiBau2sRe8Wp3J3JR0B2JKAtXNp&db=hg19&position=chr17:7578295-7578454&hgPcrResult=pack) | chr17:7578318-7578433 | TP53 | gln167-asp186 |
| GTGCTGTGACTGCTTGTAGAT | TCTGTCTCCTTCCTCTTCCTAC | [chr17:7578426+7578578](http://rohsdb.cmb.usc.edu/GBshape/cgi-bin/hgTracks?hgsid=1298971_4JiBau2sRe8Wp3J3JR0B2JKAtXNp&db=hg19&position=chr17:7578426-7578578&hgPcrResult=pack) | chr17:7578447-7578556 | TP53 | tyr126-ala161 |
| AAGTCTCATGGAAGCCAGCC | CTGGCCCCTGTCATCTTCTG | [chr17:7579276+7579417](http://rohsdb.cmb.usc.edu/GBshape/cgi-bin/hgTracks?hgsid=1298971_4JiBau2sRe8Wp3J3JR0B2JKAtXNp&db=hg19&position=chr17:7579276-7579417&hgPcrResult=pack) | chr17:7579296-7579397 | TP53 | pro98-thr125 |
| CCCAGACGGAAACCGTAGC | AGATGAAGCTCCCAGAATGCC | [chr17:7579352+7579507](http://rohsdb.cmb.usc.edu/GBshape/cgi-bin/hgTracks?hgsid=1298971_4JiBau2sRe8Wp3J3JR0B2JKAtXNp&db=hg19&position=chr17:7579352-7579507&hgPcrResult=pack) | chr17:7579371-7579486 | TP53 | glu68-gly105 |
| TCTTGGAGCCTGGGATGG | GAATGACTTTCTTACCTTCGATGC | [chr19:45855718-45855866](https://genome.ucsc.edu/cgi-bin/hgTracks?hgsid=572460167_qECOtuIyVtf5xwvaVEsRAKQNJejr&db=hg19&position=chr19:45855718-45855866&hgPcrResult=pack) | chr19:45855736-45855842 | ERCC2 | met657-lys682 |
| CAGAGAGCTCTGGGAAGACAC | AGTGACCTAACTTGCGTCTCG | chr19:45856292-45856449 | chr19:45856313-45856428 | ERCC2 | ala587-phe610 |
| CCTAGCCTCTCCCACTCACC | TGAGATCCCTCCCACTGTCC | chr19:45860509-45860657 | chr19:45860529-45860637 | ERCC2 | thr460-pro492 |
| CACCTGAGCACCGTCTTCTG | ATGCCAGCCCCTCTGAGT | [chr19:45867490+45867654](http://rohsdb.cmb.usc.edu/GBshape/cgi-bin/hgTracks?hgsid=1297053_YhBDQLfckaSyNagANWNuzEyjhKvW&db=hg19&position=chr19:45867490-45867654&hgPcrResult=pack) | chr19:45867510-45867636 | ERCC2 | asp240-leu266 |
| CATCCCTTTGGCCCCTGG | GCATGCCAATGTGGTGGTTT | [chr19:45867652-45867800](https://genome.ucsc.edu/cgi-bin/hgTracks?hgsid=572460167_qECOtuIyVtf5xwvaVEsRAKQNJejr&db=hg19&position=chr19:45867652-45867800&hgPcrResult=pack) | chr19:45867670-45867780 | ERCC2 | ser208-ile239 |
| GGAGCACCAGGATGAGTCC | GCGCTGAACCCGTAAAGG | [chr19:45872161-45872318](https://genome.ucsc.edu/cgi-bin/hgTracks?hgsid=587960663_meBubCSa4hyQfzOTnaCNVhHFDjpg&db=hg19&position=chr19:45872161-45872318&hgPcrResult=pack) | chr19:45872180-45872300 | ERCC2 | ala62-lys82 |
| CTTTACGGGTTCAGCGCATC | GAGTTTGTGTGCCCAAGGTTC | [chr19:45872302-45872466](https://genome.ucsc.edu/cgi-bin/hgTracks?hgsid=572571961_gMJYZbQm72YiP2pAGQ4hx53Kt6DU&db=hg19&position=chr19:45872302-45872466&hgPcrResult=pack) | chr19:45872322-45872445 | ERCC2 | gly36-arg61 |
| GTTAGTAGCAATGTGCCATAATAGT | GGTAATTTGGGAGCAGCAGA | [chr2:198265405+198265528](http://rohsdb.cmb.usc.edu/GBshape/cgi-bin/hgTracks?hgsid=1297593_O3lTZUTaPCQnL67b2i3SXH5i64QU&db=hg19&position=chr2:198265405-198265528&hgPcrResult=pack) | chr2:198265430-198265508 | SF3B1 | ile884-glu906 |
| GAGCAGTGAGCGGCGAG | TGTCGGCCACATAGTTCTCG | [chr2:20647153+20647362](http://rohsdb.cmb.usc.edu/GBshape/cgi-bin/hgTracks?hgsid=1298971_4JiBau2sRe8Wp3J3JR0B2JKAtXNp&db=hg19&position=chr2:20647153-20647362&hgPcrResult=pack) | chr2:20647171-20647342 | RHOB | met1-val38 |
| CAGTAAGGACGAGTTCCCCG | GGGATGTTCTCCAGCGAGTC | [chr2:20647301+20647513](http://rohsdb.cmb.usc.edu/GBshape/cgi-bin/hgTracks?hgsid=1298971_4JiBau2sRe8Wp3J3JR0B2JKAtXNp&db=hg19&position=chr2:20647301-20647513&hgPcrResult=pack) | chr2:20647321-20647493 | RHOB | val33-pro89 |
| TCATGTGCTTCTCGGTGGAC | CAGAGCACTCGAGGTAGTCG | [chr2:20647468+20647707](http://rohsdb.cmb.usc.edu/GBshape/cgi-bin/hgTracks?hgsid=1298971_4JiBau2sRe8Wp3J3JR0B2JKAtXNp&db=hg19&position=chr2:20647468-20647707&hgPcrResult=pack) | chr2:20647488-20647687 | RHOB | ser88-ala153 |
| TCCAAGCCTACGACTACCTCG | TCATAGCACCTTGCAGCAGTT | [chr2:20647678+20647817](http://rohsdb.cmb.usc.edu/GBshape/cgi-bin/hgTracks?hgsid=1298971_4JiBau2sRe8Wp3J3JR0B2JKAtXNp&db=hg19&position=chr2:20647678-20647817&hgPcrResult=pack) | chr2:20647699-20647796 | RHOB | cys159-ile190 |
| ACCCCCTCCATCAACTTCTTC | CTTCACGGTTGCCTACTGGT | [chr3:178916780+178916941](http://rohsdb.cmb.usc.edu/GBshape/cgi-bin/hgTracks?hgsid=1298971_4JiBau2sRe8Wp3J3JR0B2JKAtXNp&db=hg19&position=chr3:178916780-178916941&hgPcrResult=pack) | chr3:178916801-178916921 | PIK3CA | asp64-ile102 |
| CGCATTTCCACAGCTACACC | AGCATCAGCATTTGACTTTACCT | [chr3:178921446+178921598](http://rohsdb.cmb.usc.edu/GBshape/cgi-bin/hgTracks?hgsid=1298971_4JiBau2sRe8Wp3J3JR0B2JKAtXNp&db=hg19&position=chr3:178921446-178921598&hgPcrResult=pack) | chr3:178921466-178921575 | PIK3CA | tyr317-asp352 |
| AGGTCCATTTTAGCACTTACCTGTGAC | ACAGAGTAACAGACTAGCTAGAGAC | [chr3:178935994-178936139](http://rohsdb.cmb.usc.edu/GBshape/cgi-bin/hgTracks?hgsid=1298971_4JiBau2sRe8Wp3J3JR0B2JKAtXNp&db=hg19&position=chr3:178935994-178936139&hgPcrResult=pack) | chr3:178936019-178936115 | PIK3CA | asn521-trp552 |
| GTATGCATGCTGTTTAATTGTGTGGA | CTGGAATGCCAGAACTACAATCT | [chr3:178951968-178952143](http://rohsdb.cmb.usc.edu/GBshape/cgi-bin/hgTracks?hgsid=1297593_O3lTZUTaPCQnL67b2i3SXH5i64QU&db=hg19&position=chr3:178951968-178952143&hgPcrResult=pack) | chr3:178951992-178952120 | PIK3CA | asp1017-ile1058 |
| AGCCTCATCTAGTTTCTCGCTC | CTTCCTGCCCACCCAGACG | [chr3:184870549+184870678](http://rohsdb.cmb.usc.edu/GBshape/cgi-bin/hgTracks?hgsid=1297593_O3lTZUTaPCQnL67b2i3SXH5i64QU&db=hg19&position=chr3:184870549-184870678&hgPcrResult=pack) | chr3:184870571-184870659 | C3orf70 | met1-trp13 |
| AAGCGGCTGTTAGTCACTGG | CCTCTTCCTCAGGATTGCCTTT | [chr3:41266059+41266169](http://rohsdb.cmb.usc.edu/GBshape/cgi-bin/hgTracks?hgsid=1297593_O3lTZUTaPCQnL67b2i3SXH5i64QU&db=hg19&position=chr3:41266059-41266169&hgPcrResult=pack) | chr3:41266079-41266147 | CTNNB1 | gln26-gly48 |
| GGTTTCAGTCTCTGGATCCCAC | TGCCACTCTTAGGGTTTGGG | [chr4:153247206+153247362](http://rohsdb.cmb.usc.edu/GBshape/cgi-bin/hgTracks?hgsid=1297593_O3lTZUTaPCQnL67b2i3SXH5i64QU&db=hg19&position=chr4:153247206-153247362&hgPcrResult=pack) | chr4:153247228-153247342 | FBXW7 | ile488-lys524 |
| TCCCAACCATGACAAGATTTTCC | GTGGATCTACAGATCGGACACT | [chr4:153249332+153249471](http://rohsdb.cmb.usc.edu/GBshape/cgi-bin/hgTracks?hgsid=1297593_O3lTZUTaPCQnL67b2i3SXH5i64QU&db=hg19&position=chr4:153249332-153249471&hgPcrResult=pack) | chr4:153249355-153249449 | FBXW7 | lys444-lys472 |
| AGCTCACTGGATGTGGGGCTGTG | ATCCCTGAGCGTCATCTGCC | [chr4:1803535-1803682](http://rohsdb.cmb.usc.edu/GBshape/cgi-bin/hgTracks?hgsid=1297593_O3lTZUTaPCQnL67b2i3SXH5i64QU&db=hg19&position=chr4:1803535-1803682&hgPcrResult=pack) | chr4:1803555-1803663 | FGFR3 | glu247-asp280 |
| TTACAGGATGAACAGGAAGAAGCCC | GCCTCAACGCCCATGTCTTT | [chr4:1806033-1806145](http://rohsdb.cmb.usc.edu/GBshape/cgi-bin/hgTracks?hgsid=1297593_O3lTZUTaPCQnL67b2i3SXH5i64QU&db=hg19&position=chr4:1806033-1806145&hgPcrResult=pack) | chr4:1806053-1806123 | FGFR3 | glu360-gly380 |
| CAATGTGCTGGTGACCGAGG | TACTGGCATGACCCCCACC | [chr4:1807804+1807943](http://rohsdb.cmb.usc.edu/GBshape/cgi-bin/hgTracks?hgsid=1297593_O3lTZUTaPCQnL67b2i3SXH5i64QU&db=hg19&position=chr4:1807804-1807943&hgPcrResult=pack) | chr4:1807824-1807924 | FGFR3 | asn629-asn653 |
| CATCAGCGCTGCCTGAAACTCG | CACCCGTCCTGCCCCTT | [chr5:1295151-1295318](http://rohsdb.cmb.usc.edu/GBshape/cgi-bin/hgTracks?hgsid=1297593_O3lTZUTaPCQnL67b2i3SXH5i64QU&db=hg19&position=chr5:1295151-1295318&hgPcrResult=pack) | chr5:1295170-1295301 | TERT | PROMOTOR |
| TGACCAGGGCCTTCCTTGTA | CATTAGCGCATCACAGTCGC | [chr6:36651842+36651992](http://rohsdb.cmb.usc.edu/GBshape/cgi-bin/hgTracks?hgsid=1297593_O3lTZUTaPCQnL67b2i3SXH5i64QU&db=hg19&position=chr6:36651842-36651992&hgPcrResult=pack) | chr6:36651862-36651972 | CDKN1A | met1-ser31 |
| CCCAGTGGACAGCGAGC | GGGAAGGTAGAGCTTGGGC | [chr6:36651947+36652115](http://rohsdb.cmb.usc.edu/GBshape/cgi-bin/hgTracks?hgsid=1297593_O3lTZUTaPCQnL67b2i3SXH5i64QU&db=hg19&position=chr6:36651947-36652115&hgPcrResult=pack) | chr6:36651964-36652096 | CDKN1A | leu30-gly72 |
| GGAGGGTGACTTCGCCTGG | CAAGACAGTGACAGGTCCACA | [chr6:36652055+36652228](http://rohsdb.cmb.usc.edu/GBshape/cgi-bin/hgTracks?hgsid=1297593_O3lTZUTaPCQnL67b2i3SXH5i64QU&db=hg19&position=chr6:36652055-36652228&hgPcrResult=pack) | chr6:36652074-36652207 | CDKN1A | glu66-asp109 |
| GCACCTCACCTGCTCTGC | ATGTCCGCACCTGTCATGCT | [chr6:36652165+36652333](http://rohsdb.cmb.usc.edu/GBshape/cgi-bin/hgTracks?hgsid=1297593_O3lTZUTaPCQnL67b2i3SXH5i64QU&db=hg19&position=chr6:36652165-36652333&hgPcrResult=pack) | chr6:36652183-36652313 | CDKN1A | gln103-thr145 |
| AGCCTCAATTCTTACCATCCACA | TGAAGACCTCACAGTAAAAATAGGT | [chr7:140453060+140453180](http://rohsdb.cmb.usc.edu/GBshape/cgi-bin/hgTracks?hgsid=1297593_O3lTZUTaPCQnL67b2i3SXH5i64QU&db=hg19&position=chr7:140453060-140453180&hgPcrResult=pack) | chr7:140453083-140453155 | BRAF | asp594-ile617 |
| GCCCTCACCAGACCTGTTC | CCCGCAGGCCTAAGTCATTT | [chr9:137328287-137328469](https://genome.ucsc.edu/cgi-bin/hgTracks?hgsid=575091731_1xmnHMiitkw4CvDKcZC27yDNOnt0&db=hg19&position=chr9:137328287-137328469&hgPcrResult=pack) | chr9:137328306-137328449 | RXRA | phe415-his459 |
| AGCTGACTCATCACTGCCTACA | TGCAGAAAAGGGTCCATTGGC | [chrX:44922723+44922861](http://rohsdb.cmb.usc.edu/GBshape/cgi-bin/hgTracks?hgsid=1297593_O3lTZUTaPCQnL67b2i3SXH5i64QU&db=hg19&position=chrX:44922723-44922861&hgPcrResult=pack) | chrX:44922745-44922840 | KDM6A | asn536-leu567 |
| TTGTAGCCAATGAAGGTGCCAT | GACGGGTAGAGTGTGCGTG | [hr14:105246484+105246597](http://rohsdb.cmb.usc.edu/GBshape/cgi-bin/hgTracks?hgsid=1297593_O3lTZUTaPCQnL67b2i3SXH5i64QU&db=hg19&position=chr14:105246484-105246597&hgPcrResult=pack) | chr14:105246506-105246578 | AKT1 | gly16-asn31 |
