# Supplementary material for: Targeted deep sequencing of urothelial bladder cancers and associated urinary DNA: a 23‐gene panel with utility for non‐invasive diagnosis and risk stratification
Source: BJU Int. 2019 Jun 19;124(3):532–44. doi: 10.1111/bju.14808 (PMC6772022; doi:10.1111/bju.14808)
Supplement: Supplementary file 2 — Table S2. Mutation types and sites in bladder cancer‐associated genes. The table shows the number of moderate impact (missense mutations) and high impact mutations (start/stop gain/loss and splice site mutations) and the amino acid positions of hotspots. [file BJU-124-532-s002.docx]

**Table S2: Mutation types and sites in bladder cancer associated genes.** The table shows the number of moderate impact (missense mutations) and high impact mutations (start/stop gain/loss and splice site mutations) and the amino acid positions of hotspots.

| **Gene** | **Hotspot site** | **Impact/type** | **No. occurrences** |
| --- | --- | --- | --- |
| ***TERT*** | **chr5_1295228_G/A** | **Promoter** | **600** |
|  |  |  |  |
|  | **chr5_1295228_G/T** | **Promoter** | **14** |
|  |  |  |  |
|  | **chr5_1295250_G/A** | **Promoter** | **114** |
|  |  |  |  |
|  | **chr5_1295242_G/A &** | **Promoter** | **7** |
|  | **chr5_1295243_G/A** |  |  |
|  | ***Overall*** | **Moderate** | **459** |
| ***FGFR3*** |  | **High** | **0** |
|  | **S249** | **Missense** | **263** |
|  | **R248** | **Missense** | **56** |
|  | **G370** | **Missense** | **30** |
|  | **Y373** | **Missense** | **85** |
|  | **K650** | **Missense** | **15** |
|  | ***Overall*** | **Moderate** | **294** |
| ***PIK3CA*** |  | **High** | **0** |
|  | **E542** | **Missense** | **67** |
|  | **E545** | **Missense** | **127** |
|  | **H1047** | **Missense** | **43** |
|  | ***Overall*** | **Moderate** | **45** |
| ***HRAS*** |  | **High** | **0** |
|  | **G12/13** | **Missense** | **22** |
|  | **Q61** | **Missense** | **23** |
|  | ***Overall*** | **Moderate** | **38** |
| ***KRAS*** |  | **High** | **0** |
|  | **G12** | **Missense** | **36** |
|  | **Q61** | **Missense** | **2** |
| ***NRAS*** | ***Overall*** | **Moderate** | **9** |
|  |  | **High** | **0** |
|  | **G12** | **Missense** | **3** |
|  | **Q61** | **Missense** | **6** |
| ***TP53*** | ***Overall*** | **Moderate** | **259** |
|  |  | **High** | **58** |
|  | **R248** | **Missense** | **21** |
|  | **R273** | **Missense** | **14** |
|  | **R280** | **Missense** | **18** |
|  | **E285** | **Missense** | **23** |
| ***ERCC2*** | ***Overall*** | **Moderate** | **127** |
|  |  | **High** | **0** |
|  | **S44** | **Missense** | **10** |
|  | **N238** | **Missense** | **37** |
|  | **T484** | **Missense** | **18** |
|  | ***Overall*** | **Moderate** | **72** |
| ***RHOB*** |  | **High** | **0** |
|  | **E47** | **Missense** | **14** |
|  | **P75** | **Missense** | **11** |
|  | **E172** | **Missense** | **10** |
| ***ELF3*** | ***Overall*** | **Moderate** | **36** |
|  |  | **High** | **11** |
| ***CDKN1A*** | ***Overall*** | **Missense** | **14** |
|  |  | **Stop** | **23** |
| ***ERBB2*** | ***Overall*** | **Moderate** | **68** |
|  |  | **High** | **0** |
|  | **S310** | **Missense** | **38** |
| ***FBXW7*** | ***Overall*** | **Moderate** | **28** |
|  |  | **High** | **1** |
|  | **R465** | **Missense** | **6** |
|  | **R505** | **Missense** | **9** |
| ***RXRA*** | ***Overall*** | **Moderate** | **45** |
|  |  | **High** | **0** |
|  | **S427** | **Missense** | **40** |
| ***KDM6A*** | ***Overall*** | **Moderate** | **1** |
|  |  | **High** | **29** |
|  | **Q555*** | **Stop gain** | **27** |
| ***AKT1*** | ***Overall*** | **Moderate** | **27** |
|  |  | **High** | **0** |
|  | **E17K** | **Missense** | **27** |
| ***ERBB3*** | ***Overall*** | **Moderate** | **25** |
|  |  | **High** | **0** |
|  | **M91I** | **Missense** | **9** |
|  | **V104** | **Missense** | **12** |
| ***SF3B1*** | ***Overall*** | **Moderate** | **20** |
|  |  | **High** | **0** |
|  | **E902** | **Missense** | **14** |
|  | ***Overall*** | **Moderate** | **19** |
| ***CTNNB1*** |  | **High** | **0** |
|  | **S37** | **Missense** | **10** |
|  | **S45** | **Missense** | **9** |
| ***CREBBP*** | ***Overall*** | **Moderate** | **13** |
|  |  | **High** | **0** |
|  | **R1446** | **Missense** | **7** |
| ***BRAF*** | ***Overall*** | **Moderate** | **14** |
|  |  | **High** | **0** |
|  | **D594** | **Missense** | **6** |
|  | **V600** | **Missense** | **3** |
|  | **K601** | **Missense** | **4** |
|  | ***Overall*** | **Moderate** | **11** |
| ***C3orf70*** |  | **High** | **2** |
|  | **S6** | **Missense + stop** | **13** |
