# Supplementary material for: Targeted deep sequencing of urothelial bladder cancers and associated urinary DNA: a 23‐gene panel with utility for non‐invasive diagnosis and risk stratification
Source: BJU Int. 2019 Jun 19;124(3):532–44. doi: 10.1111/bju.14808 (PMC6772022; doi:10.1111/bju.14808)
Supplement: Supplementary file 3 — Appendix S1. Supplementary data. [file BJU-124-532-s003.docx]

**Supplementary Appendix**

**Version 3.0a**

May 9, 2019

Contents

[1 Methodology 2](#_Toc8283796)

[1.1 Overview 2](#_Toc8283797)

[1.2 Prevalence of mutations in the Cohort 2](#_Toc8283798)

[1.3 Association between mutations and survival outcomes 2](#_Toc8283799)

[1.3.1 Descriptive and univariate analyses 2](#_Toc8283800)

[1.3.2 Multivariable analyses 2](#_Toc8283801)

[2 Patient characteristics and follow-up 2](#_Toc8283802)

[2.1 Patient characteristics 2](#_Toc8283803)

[2.2 Follow-up 2](#_Toc8283804)

[3 Prevalence of mutations in the Cohort 2](#_Toc8283805)

[3.1 Mutations across type and risk groups 2](#_Toc8283806)

[3.1.1 Linear trends in mutations where tests found a statistically signiﬁcant difference 2](#_Toc8283807)

[4 Associations between mutations and survival outcomes 2](#_Toc8283808)

[4.1 Evaluable mutations 2](#_Toc8283809)

[4.2 Events 2](#_Toc8283810)

[4.3 Overall population 2](#_Toc8283811)

[4.3.1 Analyses considering the eﬀect of each gene individually 2](#_Toc8283812)

[4.3.2 Analyses which account for multiple genes 2](#_Toc8283813)

[4.4 MIBC 2](#_Toc8283814)

[4.4.1 Analyses considering the eﬀect of each gene individually 2](#_Toc8283815)

[4.4.2 Analyses which account for multiple genes 2](#_Toc8283816)

[4.5 NMIBC population 2](#_Toc8283817)

[4.5.1 Analyses considering the eﬀect of each gene individually across all NMIBC patients 2](#_Toc8283818)

[4.5.2 Analyses which account for multiple genes across all NMIBC patients 2](#_Toc8283819)

[4.6 High-risk NMIBC 2](#_Toc8283820)

List of Tables

[Table 1: Patient Characteristics 2](#_Toc8214822)

[Table 2: Median length of follow-up in years, by population 2](#_Toc8214823)

[Table 3: Comparison of the Mutations across risk groups 2](#_Toc8214824)

[Table 4: Evaluable mutations for each population 2](#_Toc8214825)

[Table 5: Events 2](#_Toc8214826)

[Table 6: Hazard ratios for the unadjusted and adjusted Cox models for the overall population 2](#_Toc8214827)

[Table 7: Hazard ratios for the unadjusted and adjusted Cox models for the MIBC population 2](#_Toc8214828)

[Table 8: Hazard ratios for the unadjusted and adjusted Cox models for the NMIBC population 2](#_Toc8214829)

[Table 9: Models for RFI with more than one mutation (adjusting for sex and risk group) 2](#_Toc8214830)

[Table 10: HR-NMIBC models 2](#_Toc8214831)

List of Figures

[Figure 1: Process of analysing the survival outcome data 2](#_Toc8214992)

[Figure 2: Kaplan-Meier curves for DSS in the entire population 2](#_Toc8214993)

[Figure 3: Kaplan-Meier curves for OS in the entire population 2](#_Toc8214994)

[Figure 4: Kaplan-Meier curves for DSS in the MIBC population 2](#_Toc8214995)

[Figure 5: Kaplan-Meier curves for OS in the MIBC population 2](#_Toc8214996)

[Figure 6: Kaplan-Meier curves for ERBB2 in the NMIBC population 2](#_Toc8214997)

[Figure 7: Kaplan-Meier curves for ERCC2 in the NMIBC population 2](#_Toc8214998)

[Figure 8: Kaplan-Meier curves for FGFR3 in the NMIBC population 2](#_Toc8214999)

[Figure 9: Kaplan-Meier curves for HRAS in the NMIBC population 2](#_Toc8215000)

[Figure 10: Kaplan-Meier curves for PIK3CA in the NMIBC population 2](#_Toc8215001)

[Figure 11: Kaplan-Meier curves for RAS in the NMIBC population 2](#_Toc8215002)

[Figure 12: Kaplan-Meier curves for RHOB in the NMIBC population 2](#_Toc8215003)

[Figure 13: Kaplan-Meier curves for RXRA in the NMIBC population 2](#_Toc8215004)

[Figure 14: Kaplan-Meier curves for TERT in the NMIBC population 2](#_Toc8215005)

[Figure 15: Kaplan-Meier curves for TP53 in the NMIBC population 2](#_Toc8215006)

[Figure 16: Kaplan-Meier curves for PFI in the HR-NMIBC population 2](#_Toc8215007)

[Figure 17: Kaplan-Meier curves for DSS in the HR-NMIBC population 2](#_Toc8215008)

# 1 Methodology

## 1.1 Overview

Clinico-pathological, mutation and outcome data were combined and used to investigate the impact, on important time-to-event outcomes, of having a mutation rather than being wild type for each of the recorded genes.

## 1.2 Prevalence of mutations in the Cohort

The EAU risk groups were calculated for patients for non muscle invasive bladder cancer (NMIBC) patients. In order to compute the risk groups for the majority of patients, the assumption was made that patients whose carcinoma-in-situ (CIS) status was given as ‘unknown’ were analogous to those where it reported no CIS. This decision was considered justiﬁable as ‘unknown’ was considered to have been recorded if the assessor could ﬁnd no signs, but was reluctant to say for deﬁnite that there was no CIS.

The number and proportion of patients with a wild type or mutated gene have been reported. Proportions were compared across type and risk group using a Chi-squared test, or Fisher’s exact test if the ﬁrst was not appropriate due to small numbers. Histograms have been produced for those genes which showed a statistically signiﬁcant association to visualise any trend.

## 1.3 Association between mutations and survival outcomes

The association between disease-speciﬁc (DSS) and overall survival (OS) were investigated for the entire population, and NMIBC and muscle invasive bladder cancer (MIBC) populations. For both survival outcomes, the time-to-event was calculated as time from the date of study entry until the date of death. Patients who were alive at the end of the study were censored at the last date they were known to be alive. For DSS, patients who died from other causes (i.e. not attributable to bladder cancer) were also censored; and patients for whom the cause of death could not be clearly classiﬁed into disease-speciﬁc or other were excluded from the analysis.

For NMIBC, two further outcomes were also examined. These were: Recurrence-free interval (RFI) and Progression-free interval (PFI). RFI was deﬁned as the time between the date of study entry and the date of recurrence. Recurrence is deﬁned as a new occurrence of bladder cancer at the same or diﬀerent site as the initial index primary cancer and excluding tumours identiﬁed at the ﬁrst check cystoscopy. PFI is deﬁned as the time between the date of trial entry and the ﬁrst known date of progression, where progression is deﬁned as upstaging to MIBC. Patients, who were not observed to have had a recurrence or progression by the time of analysis, were censored at the last date they were known to be event-free for the respective outcomes.

In order to have suﬃcient patients so that conclusions could be drawn, the following condition was imposed: at least 5% of the respective population must have the minority gene type (i.e. between 5% and 95% of the population must have a mutation).

Descriptive analysis was initially used to explore the diﬀerence between survival depending on the patient’s gene type (wild type or mutated). A Cox model was also used to formally assess this diﬀerence. These analyses were only conducted if the conditions outlined above and in Figure 1 were met. Provided there were suﬃcient events, Cox models were then ﬁtted with included inﬂuential factors in addition to one or more gene. From the Cox model analysis, a mutation was determined to be statistically signiﬁcant if p<0.05.

Figure 1 shows the process of the analysis demonstrating the conditions implemented throughout, and those leading to its termination.

**Figure 1: Process of analysing the survival outcome data**
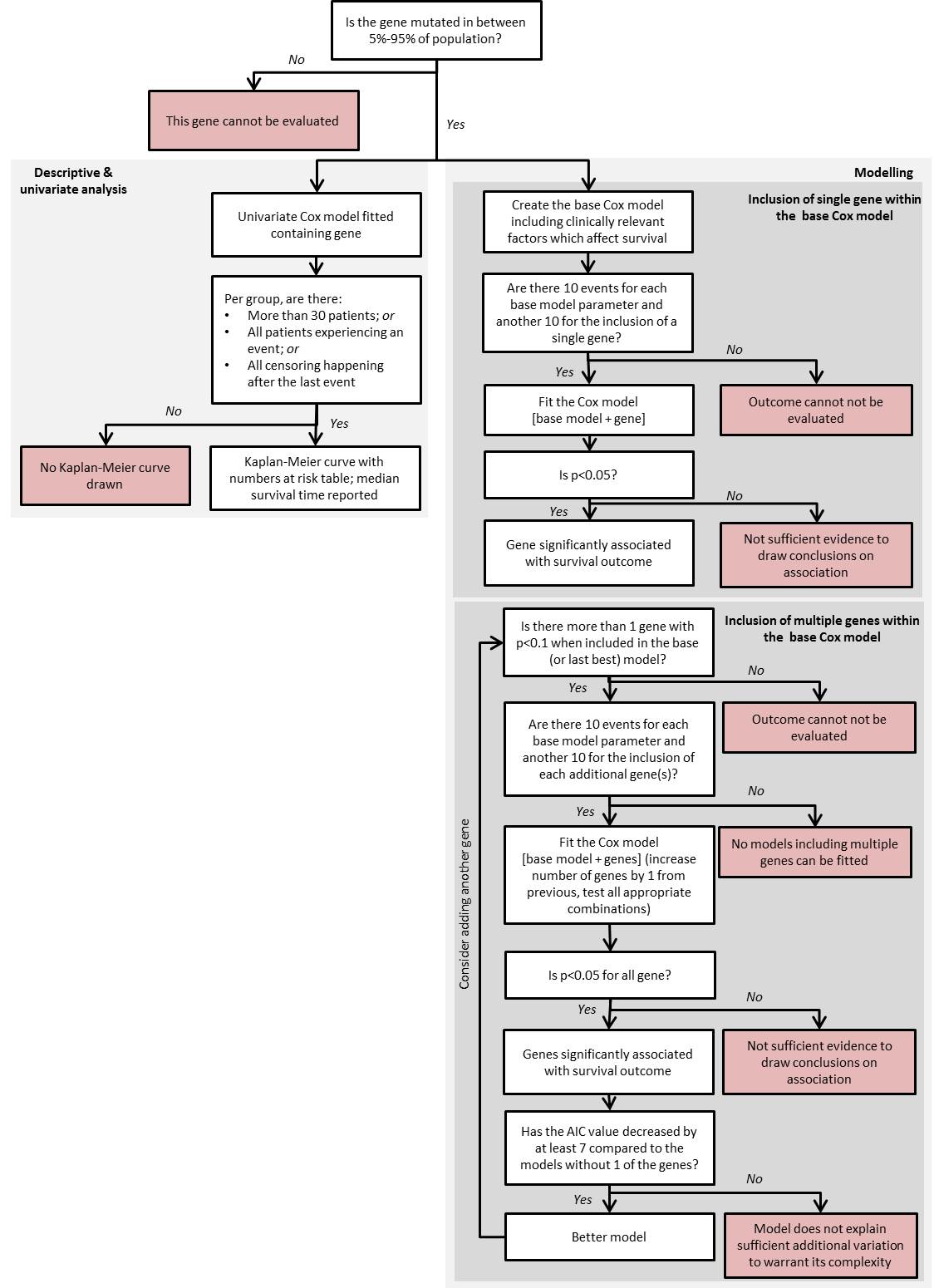


### 1.3.1 Descriptive and univariate analyses

For the entire population, Kaplan-Meier curves were produced for each gene (mutated in between 5% and 95% of the population) for DSS and OS. A ‘numbers at risk’ table and median survival time were also reported. A hazard ratio, 95% conﬁdence interval (CI) and p-value were obtained by ﬁtting a Cox model to the data, with the gene as the dependent variable. These analyses were not adjusted for any other factors (e.g. stage, grade, number of tumours, etc.). This analysis was replicated for the NMIBC population for all outcomes (RFI, PFI, DSS, OS).

It was also of interest to explore the relationship between the time-to-event outcomes and mutations in MIBC patients and in each risk group for the NMIBC. However, these analyses were subject to further conditions, since Kaplan-Meier curves for groups containing less than 30 patients can be misleading. This situation was highly likely to occur in the MIBC population as the evaluable mutation criteria meant that only 12 patients needed to have a mutation to be considered evaluable. Similarly, once stratifying by risk group, this situation was also likely in the NMIBC population. Therefore, the following decisions were made about reporting the curves in evaluable populations: where there were more than 30 patients in each stratiﬁed group (e.g. by mutation and type/risk group) the curve was reported as expected; if, instead, at least one group contained less than 30 patients this was only reported if either: 1) all patients experienced the event; or 2) all censoring times were subsequent to the maximum event time.

### 1.3.2 Multivariable analyses

Whilst the descriptive analyses can give some indication of an association, adjusted analyses were important in ensuring that any association discovered was due to the eﬀect of the mutation and not from confounding. Therefore, ‘base’ models were constructed to account for inﬂuential factors; these ‘base’ models were tailored to each population. The Cox models were also evaluated to determine whether time-dependent eﬀects were required.

For the entire population, the ‘base’ model consisted of gender and the EAU risk factors of stage, grade, number of tumours, size of largest tumour and presence of CIS. On examination, time-dependent interactions were required for the size of largest tumour for OS, and stage, grade and on some occasions CIS for DSS. The ‘base’ model for the NMIBC population adjusted for gender and patient’s risk group. The eﬀect of the risk group was found to alter over time, requiring a time-dependent eﬀect to be included. For the MIBC population, only gender was adjusted for in the ‘base’ model. To these ‘base’ models, the evaluable mutations were added singly. However, in order to ﬁt these models, it was required that there were at least 10 events for each parameter.

To further investigate the eﬀect of the mutations, models which contained more than one gene were ﬁtted, where appropriate. However, only genes which had a p-value of less than 0.1 in the adjusted single gene analysis, were considered eligible for inclusion in this. These models containing more than one gene, were ﬁtted provided that:

1. more than one mutation must have p<0.1 in the adjusted single gene analysis;

2. there were an additional 10 events (to that needed for the adjusted single gene analysis) for each additional mutation included.

Essentially an iterative process was employed to test the combinations. Where there was more than one mutation with p<0.1, initially all appropriate pairwise combinations were tested. The Akaike Information Criteria (AIC) value was also computed. Where either one or both mutations had p-values of less than 0.05, further models containing this combination would not be considered, nor would any model where the AIC value decreased by less than 7 from an similar model which excluded one of the mutations. The value of 7 was chosen as this roughly equates to the diﬀerence required in the critical value of a chi-squared distribution for a single change in the degrees of freedom. Should one model prove to be signiﬁcant (in terms of mutation p-values and change in AIC), models using an additional mutation would be considered (provided that there were suﬃcient events and mutations with p<0.1).

# 2 Patient characteristics and follow-up

## 2.1 Patient characteristics

**Table 1: Patient Characteristics**

| **Type & risk group** | **NK-NMIBC (6)** | **LR-NMIBC (88)** | **IR-NMIBC (268)** | **HR-NMIBC (370)** | **MIBC (224)** | **Overall (956)** |
| --- | --- | --- | --- | --- | --- | --- |
| **Age, years** | | | | | | |
| N | 6 | 88 | 268 | 370 | 224 | 956 |
| Median | 72.0 | 70.0 | 69.0 | 71.0 | 73.0 | 71.0 |
| Range | 47.0, 83.0 | 40.0, 89.0 | 26.0, 95.0 | 36.0, 92.0 | 35.0, 94.0 | 26.0, 95.0 |
| **Sex** (N (%)) | | | | | | |
| Male | 5 (83.3) | 65 (73.9) | 199 (74.3) | 308 (83.2) | 171 (76.3) | 748 (78.2) |
| Female | 1 (16.7) | 23 (26.1) | 69 (25.7) | 62 (16.8) | 53 (23.7) | 208 (21.8) |
| **Numbers of tumours** | | | | | | |
| N | 6 | 88 | 268 | 366 | 221 | 949 |
| Median | 1.0 | 1.0 | 1.0 | 2.0 | 1.0 | 1.0 |
| Range | 1.0, 2.0 | 1.0, 1.0 | 1.0, 10.0 | 1.0, 20.0 | 1.0, 26.0 | 1.0, 26.0 |
| **Size of largest tumour** | | | | | | |
| N | 6 | 88 | 268 | 368 | 221 | 951 |
| Median | 1.4 | 1.5 | 2.0 | 3.0 | 5.0 | 3.0 |
| Range | 0.5, 3.0 | 0.5, 2.8 | 0.4, 10.0 | 0.2, 15.0 | 0.0, 15.0 | 0.0, 15.0 |
| **Grade** (N (%)) | | | | | | |
| Grade 1 | 4 (66.7) | 88 (100.0) | 71 (26.5) | 11 ( 3.0) | 0 ( 0.0) | 174 (18.3) |
| Grade 2 | 1 (16.7) | 0 ( 0.0) | 197 (73.5) | 83 (22.6) | 9 ( 4.0) | 290 (30.4) |
| Grade 3 | 0 ( 0.0) | 0 ( 0.0) | 0 ( 0.0) | 267 (72.8) | 209 (93.3) | 476 (49.9) |
| Unable to determine | 1 (16.7) | 0 ( 0.0) | 0 ( 0.0) | 6 ( 1.6) | 6 ( 2.7) | 13 (1.4) |
| **Stage** (N (%)) | | | | | | |
| pTa | 6 (100.0) | 88 (100.0) | 268 (100.0) | 99 (26.8) | 0 ( 0.0) | 461 (48.2) |
| PTis | 0 ( 0.0) | 0 ( 0.0) | 0 ( 0.0) | 11 ( 3.0) | 0 ( 0.0) | 11 ( 1.2) |
| pT1 | 0 ( 0.0) | 0 ( 0.0) | 0 ( 0.0) | 260 (70.3) | 0 ( 0.0) | 260 (27.2) |
| pT2+ | 0 ( 0.0) | 0 ( 0.0) | 0 ( 0.0) | 0 ( 0.0) | 224 (100.0) | 224 (23.4) |
| **Presence of Carcinoma In Situ (CIS)** (N (%)) | | | | | | |
| Yes | 0 ( 0.0) | 0 ( 0.0) | 0 ( 0.0) | 96 (26.4) | 50 (22.6) | 146 (15.5) |
| No | 0 ( 0.0) | 54 (61.4) | 170 (63.4) | 165 (45.5) | 101 (45.7) | 490 (52.1) |
| Unknown | 1 (100.0) | 34 (38.6) | 98 (36.6) | 102 (28.1) | 70 (31.7) | 305 (32.4) |

This risk groups have been calculated assuming that CIS Unknown is analogous to No CIS

## 2.2 Follow-up

**Table 2: Median length of follow-up in years, by population**

| **Population** | **Median Follow-up** | **(95% CI)** |
| --- | --- | --- |
| Overall | 5.20 | (5.06, 5.29) |
| MIBC | 4.85 | (3.64, 5.39) |
| NMIBC | 5.22 | (5.11, 5.33) |
| LR-NMIBC | 4.49 | (4.10, 4.98) |
| IR-NMIBC | 5.14 | (4.86, 5.34) |
| HR-NMIBC | 5.33 | (5.20, 5.56) |

The abbreviations LR-NMIBC, IR-NMIBC and HR-NMIBC related to low risk, intermediate risk and high risk NMIBC patients respectively.

# 3 Prevalence of mutations in the Cohort

## 3.1 Mutations across type and risk groups

**Table 3: Comparison of the Mutations across risk groups**

|  | |  |  |  | **NMIBC** | | | | |  |  |
| --- | --- | --- | --- | --- | --- | --- | --- | --- | --- | --- | --- |
|  | |  | **Overall** | **All NMIBC** | **NK** | **LR** | **IR** | | **HR** | **MIBC** |  |
| **Gene, n (%)** | |  | **(n = 956)** | **(n = 732)** | **(n = 6)** | **(n= 88)** | **(n = 268)** | | **(n = 370)** | **(n = 224)** |  |
| **AKT1*^a^*** | | | | | | | | | | | |
|  | | Wild | 929 (97.2) | 707 (74.0) | 6 ( 0.6) | 81 ( 8.5) | 254 (26.6) | | 366 (38.3) | 222 (23.2) | p<0.01 |
|  | | Mutated | 27 ( 2.8) | 25 ( 2.6) | 0 ( 0.0) | 7 ( 0.7) | 14 ( 1.5) | | 4 (0.4) | 2 ( 0.2) |  |
| **BRAF*^a^*** | | | | | | | | | | | |
|  | | Wild | 943 (98.6) | 721 (75.4) | 5 ( 0.5) | 87 ( 9.1) | 267 (27.9) | | 362 (37.9) | 222 (23.2) | p =0.22 |
|  | | Mutated | 13 ( 1.4) | 11 ( 1.2) | 1 ( 0.1) | 1 ( 0.1) | 1 ( 0.1) | | 8 ( 0.8) | 2 ( 0.2) |  |
| **C3orf70*^a^*** | | | | | | | | | | | |
|  | | Wild | 943 (98.6) | 724 (75.7) | 6 ( 0.6) | 88 ( 9.2) | 265 (27.7) | | 365 (38.2) | 219 (22.9) | p =0.57 |
|  | | Mutated | 13 ( 1.4) | 8 ( 0.8) | 0 ( 0.0) | 0 ( 0.0) | 3 ( 0.3) | | 5 ( 0.5) | 5 ( 0.5) |  |
| **CDKN1A*^a^*** | | | | | | | | | | | |
|  | | Wild | 917 (95.9) | 703 (73.5) | 6 ( 0.6) | 87 ( 9.1) | 263 (27.5) | | 347 (36.3) | 214 (22.4) | p =0.02 |
|  | | Mutated | 39 ( 4.1) | 29 ( 3.0) | 0 ( 0.0) | 1 ( 0.1) | 5 ( 0.5) | | 23 ( 2.4) | 10 (1.0) |  |
| **CDKN2A*^a^*** | | | | | | | | | | | |
|  | | Wild | 946 (99.0) | 726 (75.9) | 6 ( 0.6) | 87 ( 9.1) | 267 (27.9) | | 366 (38.3) | 220 (23.0) | p =0.43 |
|  | | Mutated | 10 ( 1.0) | 6 ( 0.6) | 0 ( 0.0) | 1 ( 0.1) | 1 ( 0.1) | | 4 ( 0.4) | 4 ( 0.4) |  |
| **CREBBP*^a^*** | | | | | | | | | | | |
|  | | Wild | 943 (98.6) | 723 (75.6) | 6 ( 0.6) | 88 ( 9.2) | 264 (27.6) | | 365 (38.2) | 220 (23.0) | p =0.80 |
|  | | Mutated | 13 ( 1.4) | 9 ( 0.9) | 0 ( 0.0) | 0 ( 0.0) | 4 ( 0.4) | | 5 ( 0.5) | 4 ( 0.4) |  |
| **CTNNB1*^a^*** | | | | | | | | | | | |
|  | | Wild | 938 (98.1) | 720 (75.3) | 6 ( 0.6) | 88 ( 9.2) | 264 (27.6) | | 362 (37.9) | 218 (22.8) | p =0.46 |
|  | | Mutated | 18 ( 1.9) | 12 ( 1.3) | 0 ( 0.0) | 0 ( 0.0) | 4 ( 0.4) | | 8 ( 0.8) | 6 ( 0.6) |  |
| **ELF3*^a^*** | | | | | | | | | | | |
|  | | Wild | 912 (95.4) | 698 (73.0) | 6 ( 0.6) | 84 ( 8.8) | 263 (27.5) | | 345 (36.1) | 214 (22.4) | p =0.03 |
|  | | Mutated | 44 ( 4.6) | 34 ( 3.6) | 0 ( 0.0) | 4 ( 0.4) | 5 ( 2.6) | | 25 ( 2.6) | 10 ( 1.0) |  |
| **ERBB2*^a^*** | | | | | | | | | | | |
|  | | Wild | 891 (93.2) | 678 (70.9) | 5 ( 0.5) | 85 ( 8.9) | 257 (26.9) | | 331 (34.6) | 213 (22.3) | p<0.01 |
|  | | Mutated | 65 ( 6.8) | 54 ( 5.6) | 1 ( 0.1) | 3 ( 0.3) | 11 ( 1.2) | | 39 ( 4.1) | 11 ( 1.2) |  |
| **ERBB3*^a^*** | | | | | | | | | | | |
|  | | Wild | 933 (97.6) | 717 (75.0) | 6 ( 0.6) | 87 ( 9.1) | 263 (27.5) | | 361 (37.8) | 216 (22.6) | p =0.60 |
|  | | Mutated | 23 ( 2.4) | 15 ( 1.6) | 0 ( 0.0) | 2 ( 0.2) | 5 ( 0.5) | | 9 ( 0.9) | 8 ( 0.8) |  |
| **ERBB4*^a^*** | | | | | | | | | | | |
|  | | Wild | 954 (99.8) | 730 (76.4) | 6 ( 0.6) | 88 ( 9.2) | 266 (27.8) | | 370 (38.7) | 224 (23.4) | p =0.31 |
|  | | Mutated | 2 ( 0.2) | 2 ( 0.2) | 0 ( 0.0) | 0 ( 0.0) | 2 ( 0.2) | | 0 ( 0.0) | 0 ( 0.0) |  |
| **ERCC2*^a^*** | | | | | | | | | | | |
|  | | Wild | 819 (85.7) | 618 (64.6) | 6 ( 0.6) | 80 ( 8.4) | 238 (24.9) | | 294 (30.8) | 201 (21.0) | p<0.01 |
|  | | Mutated | 137 (14.3) | 114 (11.9) | 0 ( 0.0) | 8 ( 0.8) | 30 (3.1) | | 76 ( 7.9) | 23 ( 2.4) |  |
| **FBXW7*^a^*** | | | | | | | | | | | |
|  | | Wild | 930 (97.3) | 713 (74.6) | 6 ( 0.6) | 86 ( 9.0) | 264 (27.6) | | 357 (37.3) | 217 (22.7) | p =0.45 |
|  | | Mutated | 26 ( 2.7) | 19 ( 2.0) | 0 ( 0.0) | 2 ( 0.2) | 4 ( 0.4) | | 13 ( 1.4) | 7 ( 0.7) |  |
| **FGFR3** | | | | | | | | | | | |
|  | | Wild | 526 (55.0) | 331 (34.6) | 2 ( 0.2) | 27 ( 2.8) | 71 ( 7.4) | | 231 (24.2) | 195 (20.4) | p<0.01 |
|  | | Mutated | 430 (45.0) | 401 (41.9) | 4 ( 0.4) | 61 ( 6.4) | 197 (20.6) | | 139 (14.5) | 29 ( 3.0) |  |
| **HRAS*^a^*** | | | | | | | | | | | |
|  | | Wild | 911 (95.3) | 693 (72.5) | 5 ( 0.5) | 81 ( 8.5) | 253 (26.5) | | 354 (37.0) | 218 (22.8) | p =0.17 |
|  | | Mutated | 45 ( 4.7) | 39 ( 4.1) | 1 ( 0.1) | 7 ( 0.7) | 15 ( 1.6) | | 16 ( 1.7) | 6 ( 0.6) |  |
| **KDM6A*^a^*** | | | | | | | | | | | |
|  | | Wild | 926 (96.9) | 707 (74.0) | 6 ( 0.6) | 86 ( 9.0) | 257 (26.9) | | 358 (37.4) | 219 (22.9) | p =0.70 |
|  | | Mutated | 30 ( 3.1) | 25 ( 2.6) | 0 ( 0.0) | 2 ( 0.2) | 11 ( 1.2) | | 12 ( 1.3) | 5 ( 0.5) |  |
| **KRAS*^a^*** | | | | | | | | | | | |
|  | | Wild | 919 (96.1) | 703 (73.5) | 6 ( 0.6) | 80 ( 8.4) | 265 (27.7) | | 352 (36.8) | 216 (22.6) | p<0.01 |
|  | | Mutated | 37 ( 3.9) | 29 ( 3.0) | 0 ( 0.0) | 8 ( 0.8) | 3 ( 0.3) | | 18 ( 1.9) | 8 ( 0.8) |  |
| **NRAS*^a^*** | | | | | | | | | | | |
|  | | Wild | 947 (99.1) | 724 (75.7) | 6 ( 0.6) | 88 ( 9.2) | 264 (27.6) | | 366 (38.3) | 223 (23.3) | p =0.67 |
|  | | Mutated | 9 ( 0.9) | 8 ( 0.8) | 0 ( 0.0) | 0 ( 0.0) | 4 ( 0.4) | | 4 ( 0.4) | 1 (0.1) |  |
| **PIK3CA** | | | | | | | | | | | |
|  | | Wild | 650 (68.0) | 494 (51.7) | 4 ( 0.4) | 51 ( 5.3) | 165 (17.3) | | 274 (28.7) | 156 (16.3) | p<0.01 |
|  | | Mutated | 306 (32.0) | 238 (24.9) | 2 ( 0.2) | 37 ( 3.9) | 103 (10.8) | | 96 (10.0) | 68 ( 7.1) |  |
| **RHOB*^a^*** | | | | | | | | | | | |
|  | | Wild | 885 (92.6) | 675 (70.6) | 6 ( 0.6) | 80 ( 8.4) | 247 (25.8) | | 342 (35.8) | 210 (22.0) | p =0.81 |
|  | | Mutated | 71 ( 7.4) | 57 ( 6.0) | 0 ( 0.0) | 8 ( 0.8) | 21 ( 2.2) | | 28 ( 2.9) | 14 ( 1.5) |  |
| **RXRA*^a^*** | | | | | | | | | | | |
|  | | Wild | 911 (95.3) | 695 (72.7) | 6 ( 0.6) | 87 ( 9.1) | 257 (26.9) | | 345 (36.1) | 216 (22.6) | p =0.10 |
|  | | Mutated | 45 ( 4.7) | 37 ( 3.9) | 0 ( 0.0) | 1 ( 0.1) | 11 ( 1.2) | | 25 ( 2.6) | 8 ( 0.8) |  |
| **SF3B1*^a^*** | | | | | | | | | | | |
|  | | Wild | 936 (97.9) | 720 (75.3) | 6 ( 0.6) | 88 ( 9.2) | 265 (27.7) | | 361 (37.8) | 216 (22.6) | p =0.14 |
|  | | Mutated | 20 ( 2.1) | 12 ( 1.3) | 0 ( 0.0) | 0 ( 0.0) | 3 ( 0.3) | | 9 ( 0.9) | 8 ( 0.8) |  |
| **TERT** | | | | | | | | | | | |
|  | | Wild | 223 (23.3) | 194 (20.3) | 1 ( 0.1) | 37 ( 3.9) | 74 ( 7.7) | | 82 ( 8.6) | 29 ( 3.0) | p<0.01 |
|  | | Mutated | 733 (76.7) | 538 (56.3) | 5 ( 0.5) | 51 ( 5.3) | 194 (20.3) | | 288 (30.1) | 195 (20.4) |  |
| **TP53*^a^*** | | | | | | | | | | | |
|  | | Wild | 694 (72.6) | 590 (61.7) | 6 ( 0.6) | 83 ( 8.7) | 246 (25.7) | | 255 (26.7) | 104 (10.9) | p<0.01 |
|  | | Mutated | 262 (27.4) | 142 (14.9) | 0 ( 0.0) | 5 ( 0.5) | 22 ( 2.3) | | 115 (12.0) | 120 (12.6) |  |
| **RAS*^a^*** | | | | | | | | | | | |
|  | | Wild | 867 (90.7) | 658 (68.8) | 5 ( 0.5) | 73 ( 7.6) | 247 (25.8) | | 333 (34.8) | 209 (21.9) | p =0.04 |
|  | | Mutated | 89 ( 9.3) | 74 ( 7.7) | 1 ( 0.1) | 15 ( 1.6) | 21 ( 2.2) | | 37 (3.9) | 15 (1.6) |  |
| *^a^* | Fishers exact test employed to test for differences as numbers too small for χ^2^ | | | | | | |  |  |  |  |
| NK | Not known: NMIBC risk group could not be calculated | | | | | | |  |  |  |  |
| LR | Low Risk (NMIBC) | | | | | | |  |  |  |  |
| IR | Intermediate Risk (NMIBC) | | | | | | |  |  |  |  |
| HR | High Risk (NMIBC) | | | | | | |  |  |  |  |
| RAS | Patient has at least 1 mutated RAS gene (HRAS, KRAS, NRAS) | | | | | | |  |  |  |  |

### 3.1.1 Linear trends in mutations where tests found a statistically signiﬁcant difference

For some of the mutations where the tests found a statistically signiﬁcant difference between the type/risk groups and wild or mutated gene, there is a slight linear trend in the number of patients for each group. These are shown in Figure 2 of the main text.

**Upwards trend with increasing severity**

For two genes, **TERT** and **TP53** the proportion of patients with mutated genes increases as the severity increases (e.g. higher proportions for IR-NMIBC compared to LR-NMIBC, higher proportions for HR-NMIBC compared to IR-NMIBC, and higher proportions for MIBC compared to HR-NMIBC).

**Downwards trend with increasing severity**

For **AKT1**, the proportion of patients with mutated genes decreases as the severity increases (e.g. lower proportions for IR-NMIBC compared to LR-NMIBC, etc.).

**Upwards trend with increasing severity within the NMIBC population**

For three genes, **CDKN1A**, **ERBB2** and **ERCC2** the proportion of patients with mutated genes increases as the risk group increases (e.g. higher proportions for IR-NMIBC compared to LR-NMIBC and higher proportions for HR-NMIBC compared to IR-NMIBC). However, the proportion of patients with a mutation and MIBC disease is not higher than that for HR-NMIBC.

**Downwards trend with increasing severity within the NMIBC population**

For **PIK3CA** the proportion of patients with mutated genes decreases as the risk group increases (e.g. lower proportions for IR-NMIBC compared to LR-NMIBC and lower proportions for HR-NMIBC compared to IR-NMIBC). However, the proportion of patients with a mutation and MIBC disease is not lower than that for HR-NMIBC.

The proportion of patients with mutated **FGFR3** was considerably higher for LR-NMIBC and IR-NMIBC patients than in the HR-NMIBC and MIBC groups. For **ELF3**, **KRAS** and **RAS** there is no clear trend.

# 4 Associations between mutations and survival outcomes

## 4.1 Evaluable mutations

To ensure that there are suﬃcient patients with a mutation, only genes where at least 5% and no more than 95% of patients have a mutated gene will be considered as ‘evaluable’ for the prognostic models.

The NMIBC population has been considered diﬀerently to the overall and MIBC populations. It looks at those mutations where the prevalence in whole NMIBC population is between 5% and 95% and also where one or more of the risk groups has a proportion above the 5% threshold.

**Table 4: Evaluable mutations for each population**

| **Population** | **Evaluable mutations** |
| --- | --- |
| Overall population: | ERBB2, ERCC2, FGFR3, PIK3CA, RAS, RHOB, TERT, TP53 |
| NMIBC population: | ERBB2, ERCC2, FGFR3, HRAS, PIK3CA, RAS, RHOB, RXRA, TERT, TP53 |
| MIBC population: | ERCC2, FGFR3, PIK3CA, RAS, RHOB, TERT, TP53 |

## 4.2 Events

**Table 5: Events**

| **Outcome** | **Overall (916)** | **NMIBC (726)** | **MIBC (227)** |
| --- | --- | --- | --- |
| **RFI status** (N (%)) | | | |
| Recurrence-free | 0 ( 0.0) | 437 (60.2) | 0 ( 0.0) |
| Experienced recurrence | 0 (0.0) | 289 (39.8) | 0 ( 0.0) |
| n/a for this population | 916 (100.0) | 0 ( 0.0) | 227 (100.0) |
| **Total** | 916 (100.0) | 726 (100.0) | 227 (100.0) |
| **PFI status** (N (%)) | | | |
| Progression-free | 0 ( 0.0) | 701 (96.6) | 0 ( 0.0) |
| Progressed to T2+ | 0 ( 0.0) | 25 ( 3.4) | 0 ( 0.0) |
| n/a for this population | 916 (100.0) | 0 ( 0.0) | 227 (100.0) |
| **Total** | 916 (100.0) | 726 (100.0) | 227 (100.0) |
| **DSS status** (N (%)) | | | |
| Censored | 776 (84.7) | 680 (93.7) | 121 (53.3) |
| Dead from bladder cancer | 131 (14.3) | 41 ( 5.6) | 102 (44.9) |
| Excluded: cause of death not reported | 9 ( 1.0) | 5 ( 0.7) | 4 ( 1.8) |
| **Total** | 916 (100.0) | 726 (100.0) | 227 (100.0) |
| **OS status** (N (%)) | | | |
| Alive | 578 (63.1) | 534 (73.6) | 61 (26.9) |
| Dead | 338 (36.9) | 192 (26.4) | 166 (73.1) |
| **Total** | 916 (100.0) | 726 (100.0) | 227 (100.0) |

## 4.3 Overall population

### 4.3.1 Analyses considering the eﬀect of each gene individually

**Figure 2: Kaplan-Meier curves for DSS in the entire population**

***
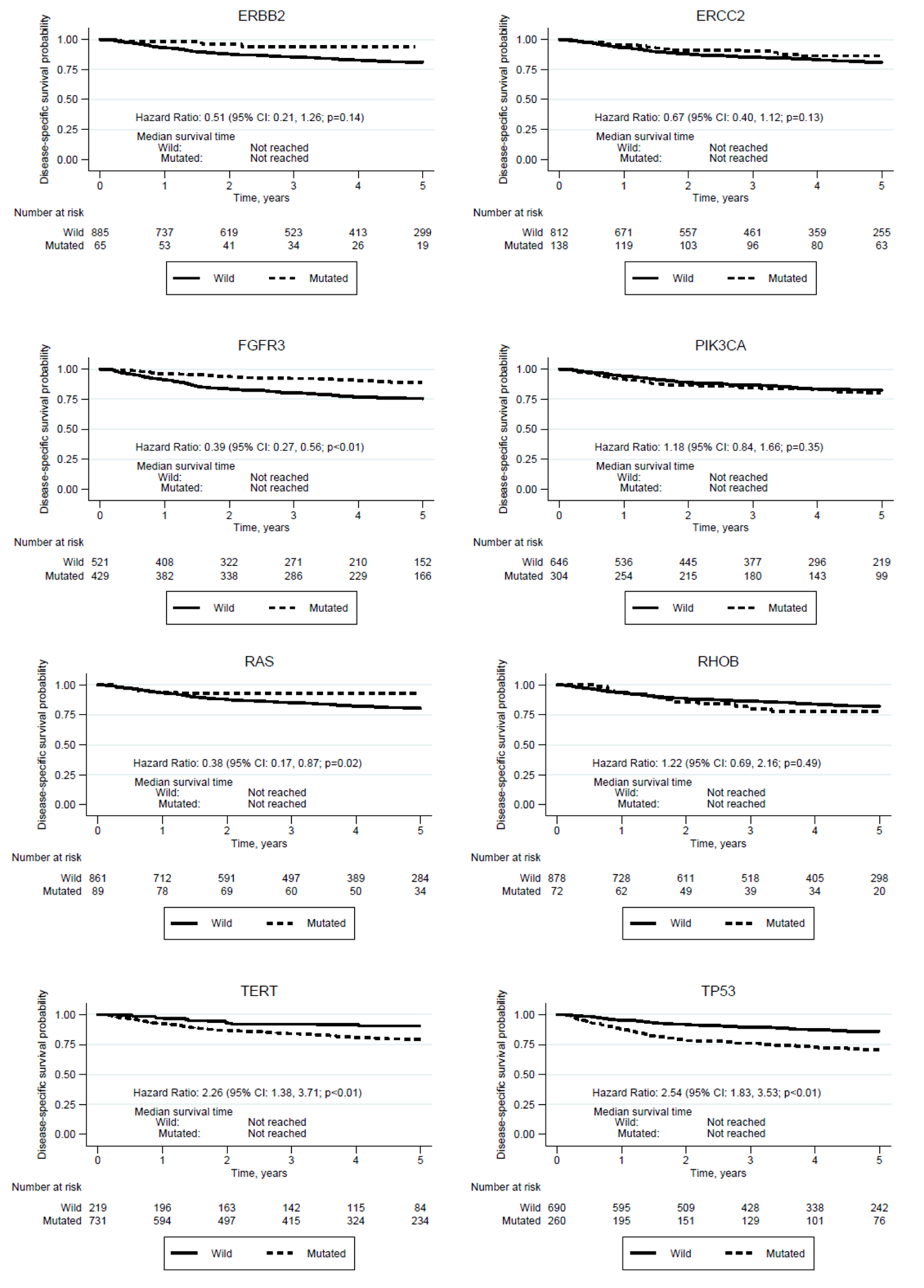
***

**Figure 3: Kaplan-Meier curves for OS in the entire population**

**
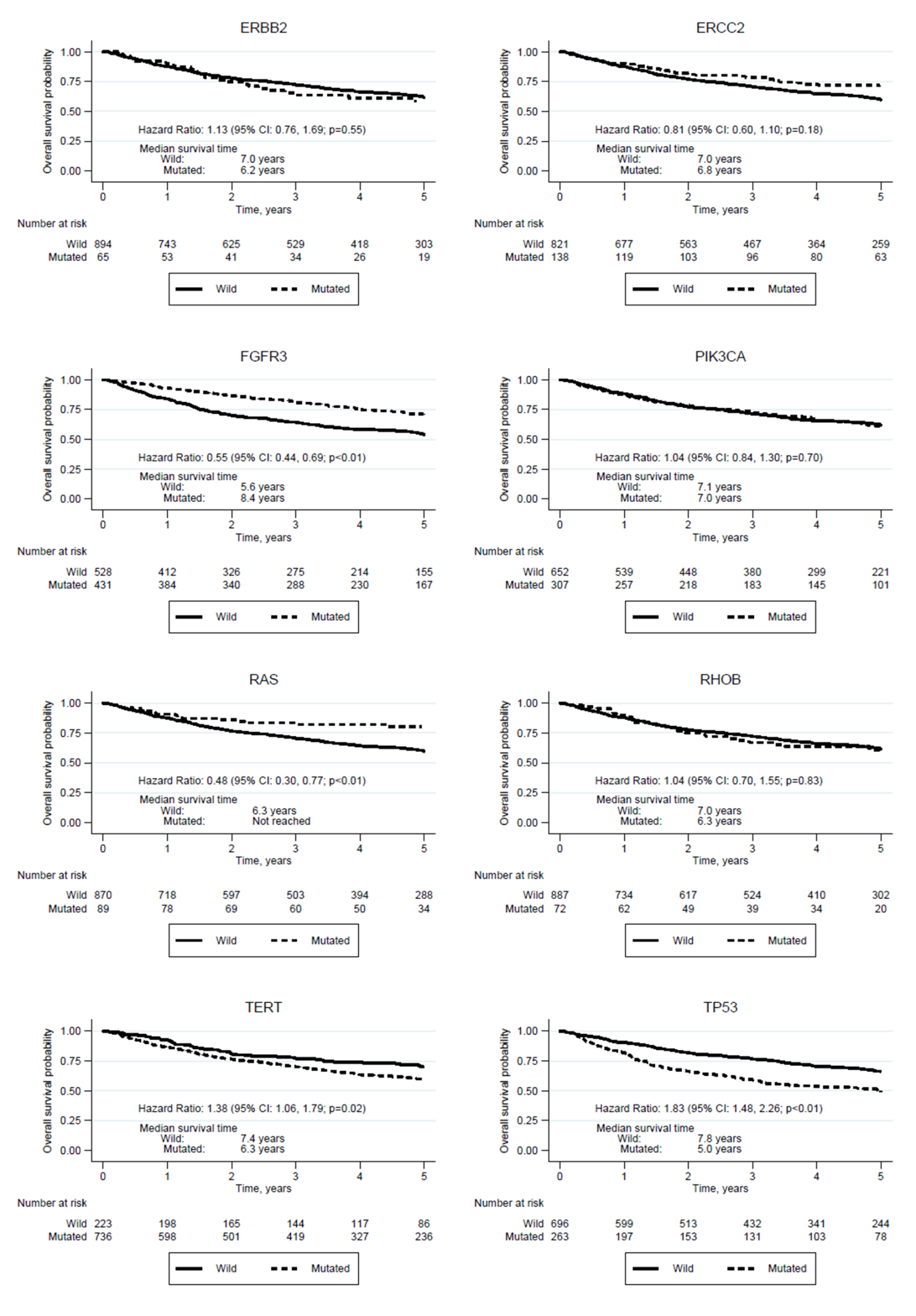
**

**Table 6: Hazard ratios for the unadjusted and adjusted Cox models for the overall population**

|  | **Mutation** | **Unadjusted model results**  gene incl. as only covariate | | | **Additional covariates in**  **adjusted model** | | **Adjusted model results** | | |
| --- | --- | --- | --- | --- | --- | --- | --- | --- | --- |
| DSS | ERBB2 | 0.51 | (95% CI: 0.21, 1.26); | p=0.14 | incl. | sex; | Insufficient events to fit model | | |
|  | ERCC2 | 0.67 | (95% CI: 0.40, 1.12); | p=0.13 |  | stage (varies over time); |  |  |  |
|  | FGFR3 | 0.39 | (95% CI: 0.27, 0.56); | p<0.01 |  | grade (varies over time); |  |  |  |
|  | PIK3CA | 1.18 | (95% CI: 0.84, 1.66); | p=0.35 |  | number of tumours; |  |  |  |
|  | RAS | 0.38 | (95% CI: 0.17, 0.87); | p=0.02 |  | size of largest tumour; |  |  |  |
|  | RHOB | 1.22 | (95% CI: 0.69, 2.16); | p=0.49 |  | presence of carcinoma-in situ |  |  |  |
|  | TERT | 2.26 | (95% CI: 1.38, 3.71); | p<0.01 |  | (varies over time for some models). |  |  |  |
|  | TP53 | 2.54 | (95% CI: 1.83, 3.53); | p<0.01 |  |  |  |  |  |
| OS | ERBB2 | 1.13 | (95% CI: 0.76, 1.69); | p=0.55 | incl. | sex; | 1.20 | (95% CI: 0.78, 1.83); | p=0.41 |
|  | ERCC2 | 0.81 | (95% CI: 0.60, 1.10); | p=0.18 |  | stage; | 0.89 | (95% CI: 0.64, 1.22); | p=0.46 |
|  | FGFR3 | 0.55 | (95% CI: 0.44, 0.69); | p<0.01 |  | grade; | 1.09 | (95% CI: 0.83, 1.43); | p=0.54 |
|  | PIK3CA | 1.04 | (95% CI: 0.84, 1.30); | p=0.70 |  | number of tumours; | 1.09 | (95% CI: 0.87, 1.38); | p=0.46 |
|  | RAS | 0.48 | (95% CI: 0.30, 0.77); | p<0.01 |  | size of largest tumour | 0.60 | (95% CI: 0.37, 0.97); | p=0.04 |
|  | RHOB | 1.04 | (95% CI: 0.70, 1.55); | p=0.83 |  | (varies over time); | 1.05 | (95% CI: 0.70, 1.59); | p=0.81 |
|  | TERT | 1.38 | (95% CI: 1.06, 1.79); | p=0.02 |  | presence of carcinoma-in situ. | 1.06 | (95% CI: 0.80, 1.40); | p=0.68 |
|  | TP53 | 1.83 | (95% CI: 1.48, 2.26); | p<0.01 |  |  | 0.96 | (95% CI: 0.75, 1.24); | p=0.76 |

The base model for DSS in the entire population was created but when this, alongside individual mutations, was tested for non-proportional hazards, stage and grade were found to violate the proportional hazard (PH) assumption (p< 0.05). For some of the mutation models, the presence of CIS was also found to violate the PH assumption as well. Therefore, any ﬁnal models must account for these violations. Unfortunately, to adjust for these time-dependent eﬀects more parameters are required, and this meant that the condition of 10 events per parameter was not met. Thus, no adjusted results for DSS are reported.

For OS, only RAS was found to be statistically signiﬁcant (p = 0.04) in the entire population, after accounting for the patient’s sex and the ﬁve EAU risk factors (listed in the table above). This ﬁnding suggests that the all-cause mortality rate in patients with a RAS mutation is reduced by 40% (HR: 0.60 (95% CI: 0.37, 0.97)) compared to those with wild type RAS.

### 4.3.2 Analyses which account for multiple genes

No models which included more than one gene were considered. For DSS, this was because of insuﬃcient events to ﬁt the base model with even just one mutation. For OS, this was because there was only one gene that met the p< 0.1 criteria.

## 4.4 MIBC

### 4.4.1 Analyses considering the eﬀect of each gene individually

**Figure 4: Kaplan-Meier curves for DSS in the MIBC population**


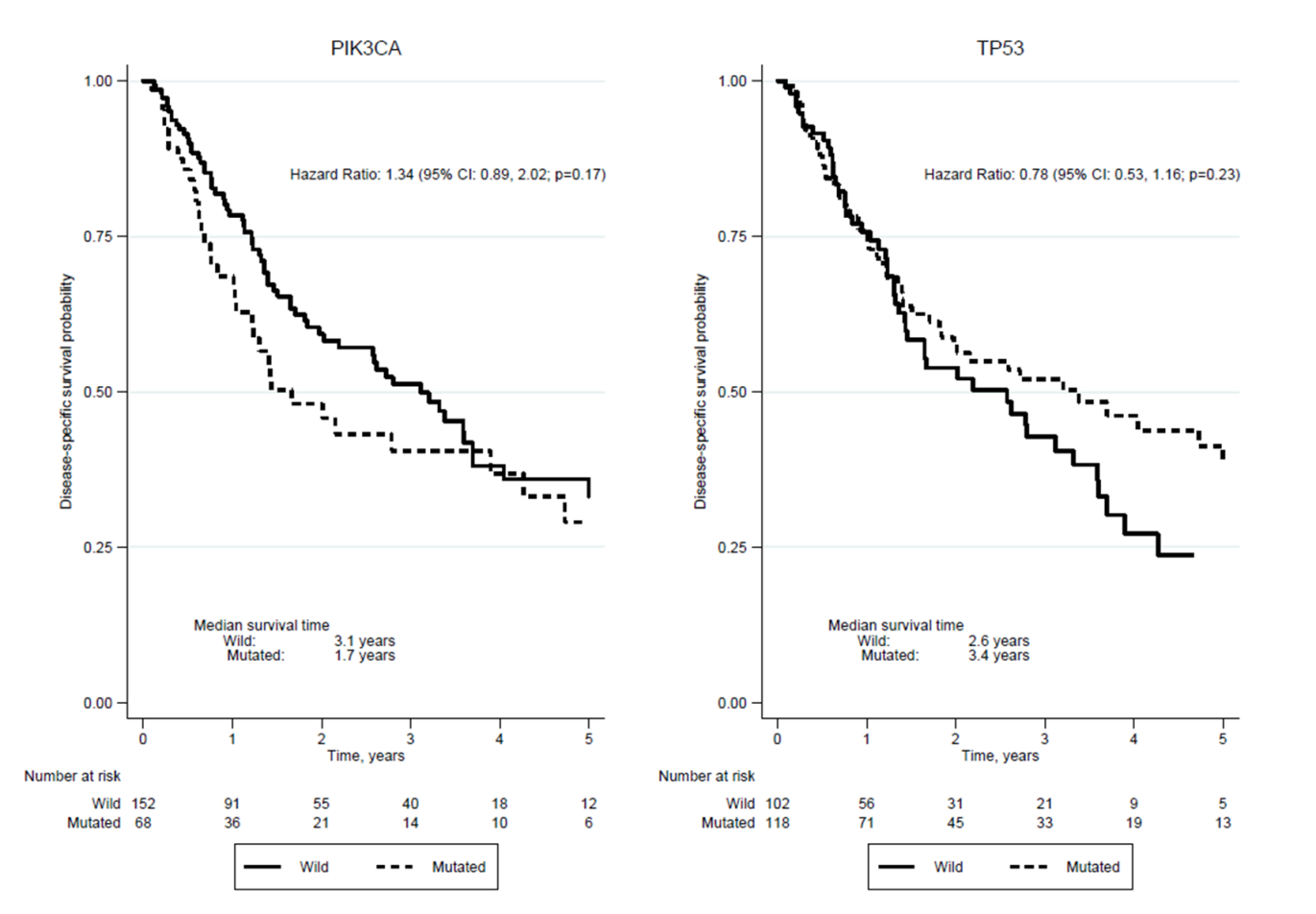


**Figure 5: Kaplan-Meier curves for OS in the MIBC population**


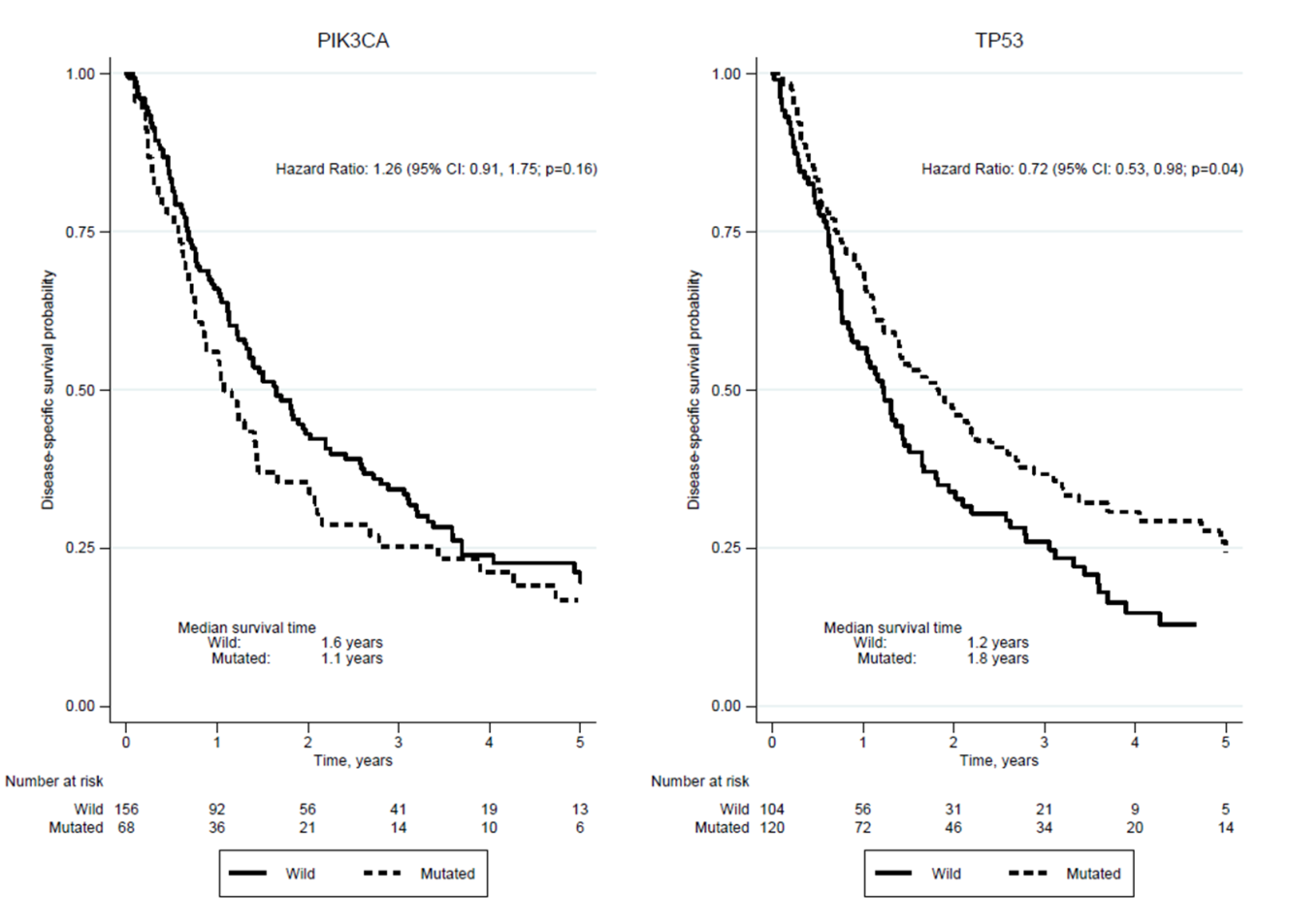


Please note that each row of Table 7 corresponds to a diﬀerent model.

**Table 7: Hazard ratios for the unadjusted and adjusted Cox models for the MIBC population**

|  | **Mutation** | **Unadjusted model results**  gene incl. as only covariate | | | |  | |  | | **Adjusted model results**  accounts for the sex in addition to gene | | | | |
| --- | --- | --- | --- | --- | --- | --- | --- | --- | --- | --- | --- | --- | --- | --- |
| DSS | ERCC2 | 0.89 | (95% CI: 0.47, 1.66); | | p=0.71 |  | |  | | 0.90 | | (95% CI: 0.48, 1.68); | | p=0.73 |
|  | FGFR3 | 1.77 | (95% CI: 1.06, 2.95); | | p=0.03 |  | |  | | 1.76 | | (95% CI: 1.05, 2.93); | | p=0.03 |
|  | PIK3CA | 1.34 | (95% CI: 0.89, 2.02); | | p=0.17 |  | |  | | 1.33 | | (95% CI: 0.88, 2.01); | | p=0.17 |
|  | RAS | 0.94 | (95% CI: 0.41, 2.16); | | p=0.89 |  | |  | | 0.95 | | (95% CI: 0.42, 2.17); | | p=0.90 |
|  | RHOB | 1.04 | (95% CI: 0.48, 2.25); | | p=0.92 |  | |  | | 1.09 | | (95% CI: 0.50, 2.38); | | p=0.83 |
|  | TERT | 1.30 | (95% CI: 0.68, 2.51); | | p=0.43 |  | |  | | 1.38 | | (95% CI: 0.71, 2.69); | | p=0.34 |
|  | TP53 | 0.78 | (95% CI: 0.53, 1.16); | | p=0.23 |  | |  | | 0.79 | | (95% CI: 0.53, 1.17); | | p=0.24 |
| OS | ERCC2 | 0.83 | (95% CI: 0.50, 1.38); | p=0.48 | | |  | |  | | 0.84 | | (95% CI: 0.51, 1.39); | p=0.50 |
|  | FGFR3 | 1.51 | (95% CI: 0.98, 2.32); | p=0.06 | | |  | |  | | 1.50 | | (95% CI: 0.97, 2.30); | p=0.07 |
|  | PIK3CA | 1.26 | (95% CI: 0.91, 1.75); | p=0.16 | | |  | |  | | 1.26 | | (95% CI: 0.91, 1.74); | p=0.17 |
|  | RAS | 0.91 | (95% CI: 0.48, 1.73); | p=0.77 | | |  | |  | | 0.92 | | (95% CI: 0.48, 1.75); | p=0.80 |
|  | RHOB | 0.90 | (95% CI: 0.47, 1.70); | p=0.74 | | |  | |  | | 0.93 | | (95% CI: 0.49, 1.78); | p=0.83 |
|  | TERT | 1.00 | (95% CI: 0.63, 1.58); | p=1.00 | | |  | |  | | 1.05 | | (95% CI: 0.65, 1.67); | p=0.85 |
|  | TP53 | 0.72 | (95% CI: 0.53, 0.98); | p=0.04 | | |  | |  | | 0.73 | | (95% CI: 0.53, 0.99); | p=0.04 |

Whilst checking the model assumptions for DSS, there was some indication that the eﬀect of RAS was non-proportional (p=0.03). However, when a time-dependent eﬀect was included, neither main or time dependent eﬀects for RAS were statistically signiﬁcant at the 5% level (main eﬀect, p = 0.60; time dependent eﬀect, p = 0.08).

Of all the univariate analyses for DSS, FGFR3 was found to have statistically signiﬁcant association with DSS in the MIBC population. The disease-speciﬁc mortality rate in patients with a mutation was 77% higher than for those with wild type FGFR3.

From the table above, it can be seen that TP53 mutation is statistically signiﬁcantly associated with OS in the MIBC population at the 5% level, suggesting a 27% reduction in the mortality rate for those with wild type TP53 compared to mutated TP53.

### 4.4.2 Analyses which account for multiple genes

Models which included more than one gene were only considered if p< 0.1 for more than one gene. For DSS, this condition was not met.

In Table 7, it can be seen that both TP53 and FGFR3 mutations have p-values of less than 0.1 for OS in the MIBC population. Therefore, another Cox model was ﬁtted, which contained sex, TP53 and FGFR3 as covariates. In this model, the hazard ratio (95% CI) for FGFR3 is 1.323 (0.839, 2.086) and TP53 0.771 (0.556, 1.071). Since neither mutation was statistically signiﬁcant in this model, there is not suﬃcient evidence to conclude whether these mutations both aﬀect OS in the MIBC population.

## 4.5 NMIBC population

### 4.5.1 Analyses considering the eﬀect of each gene individually across all NMIBC patients

**Figure 6: Kaplan-Meier curves for ERBB2 in the NMIBC population**


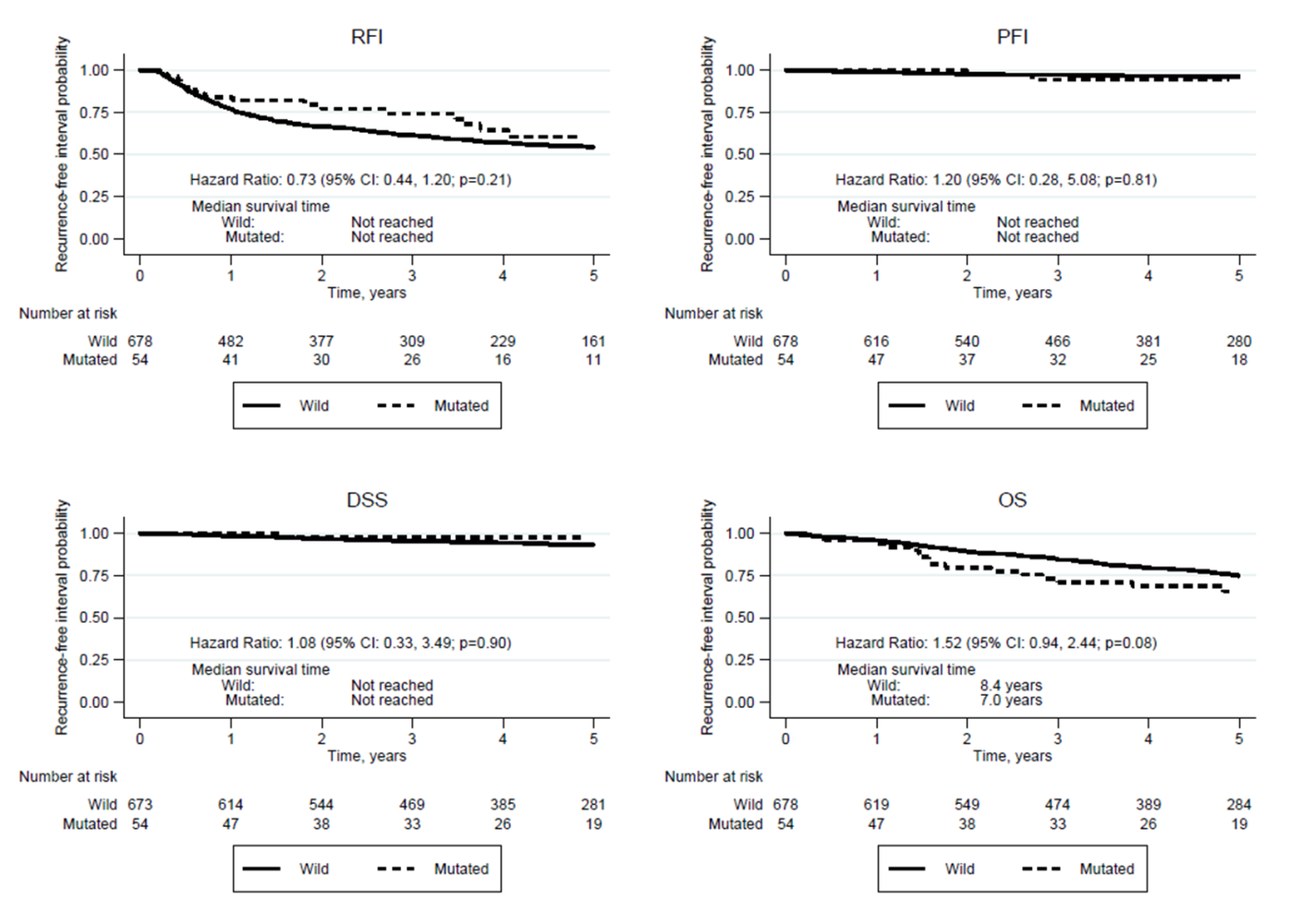


**Figure 7: Kaplan-Meier curves for ERCC2 in the NMIBC population**


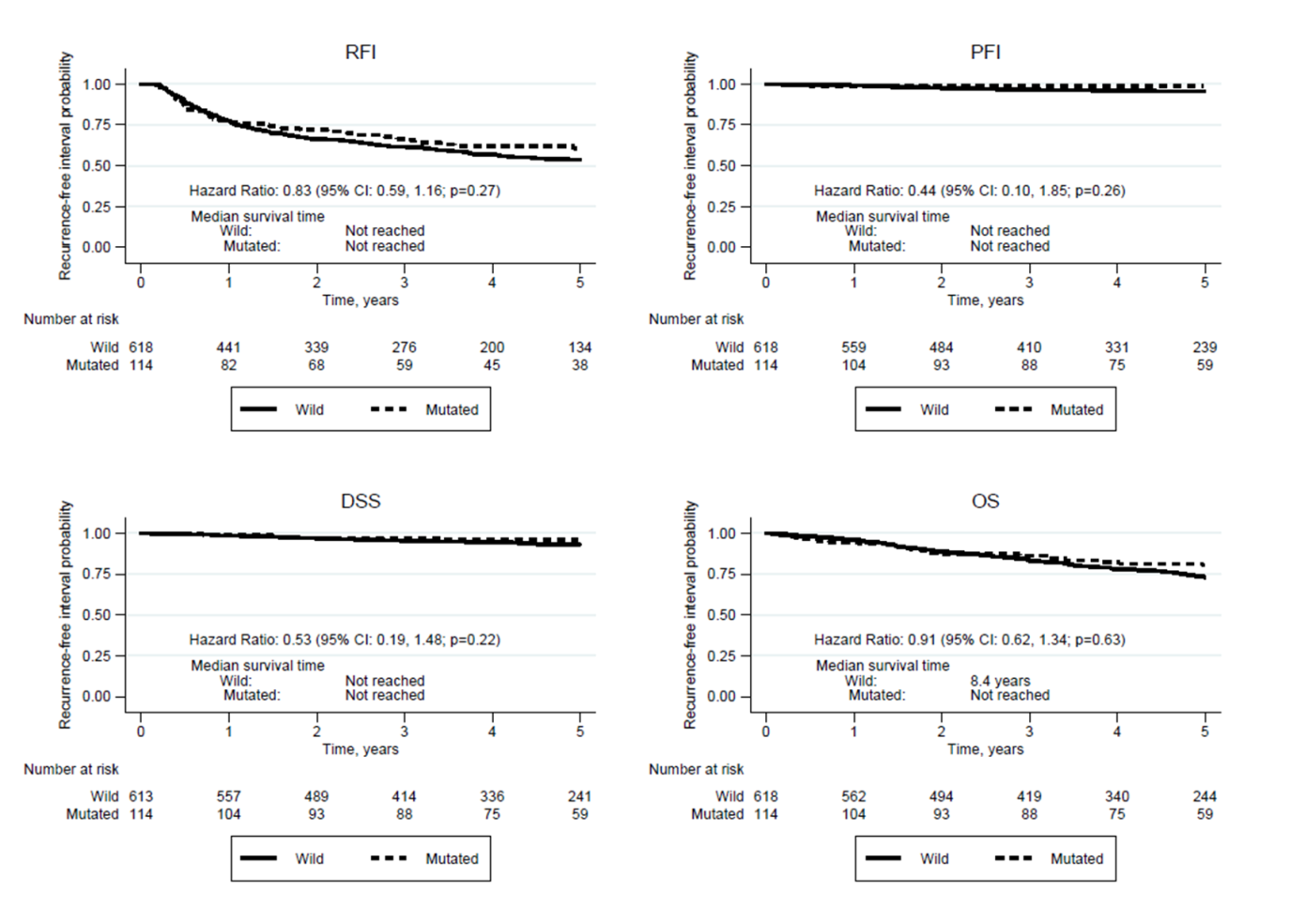


**Figure 8: Kaplan-Meier curves for FGFR3 in the NMIBC population**


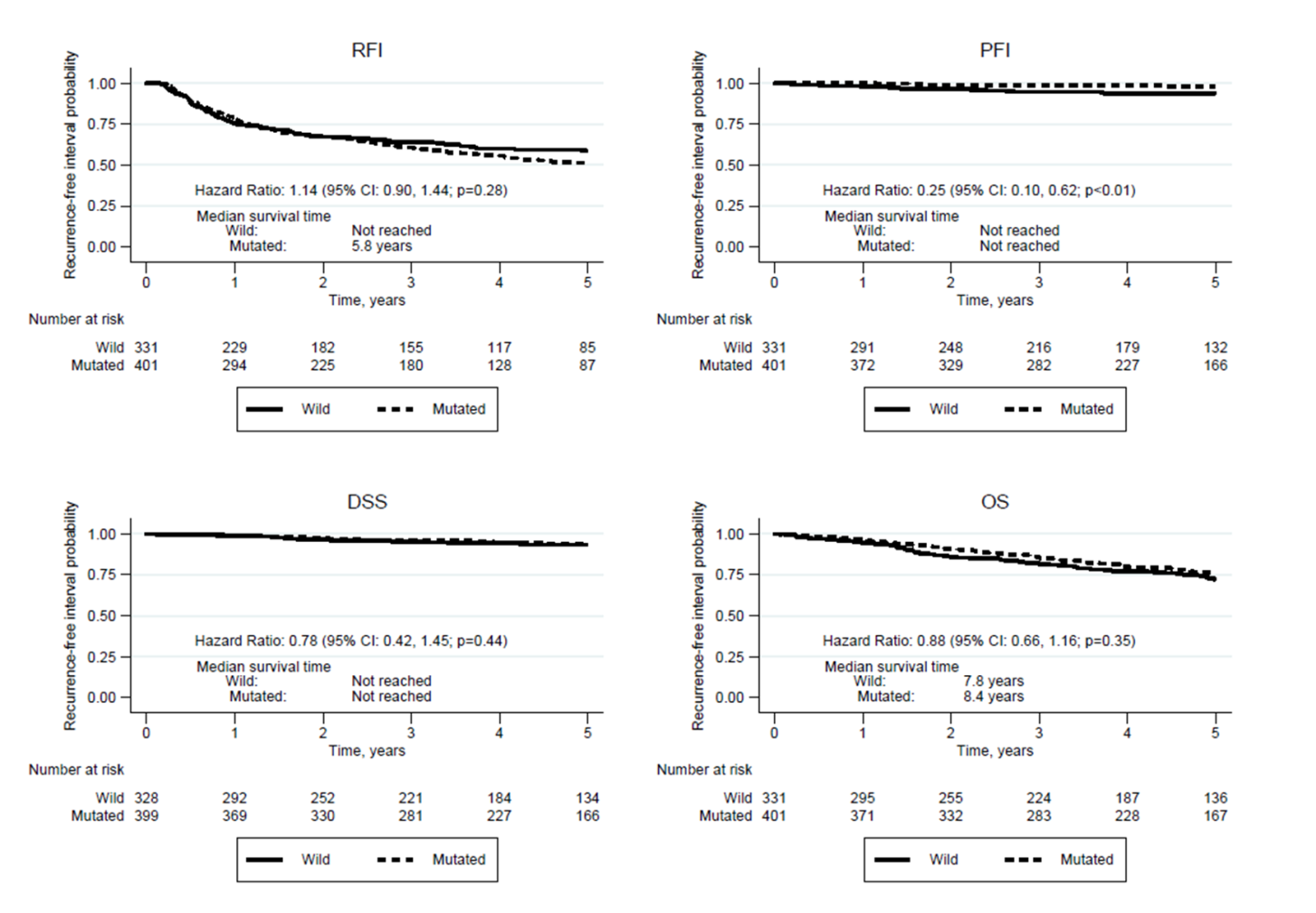


**Figure 9: Kaplan-Meier curves for HRAS in the NMIBC population**


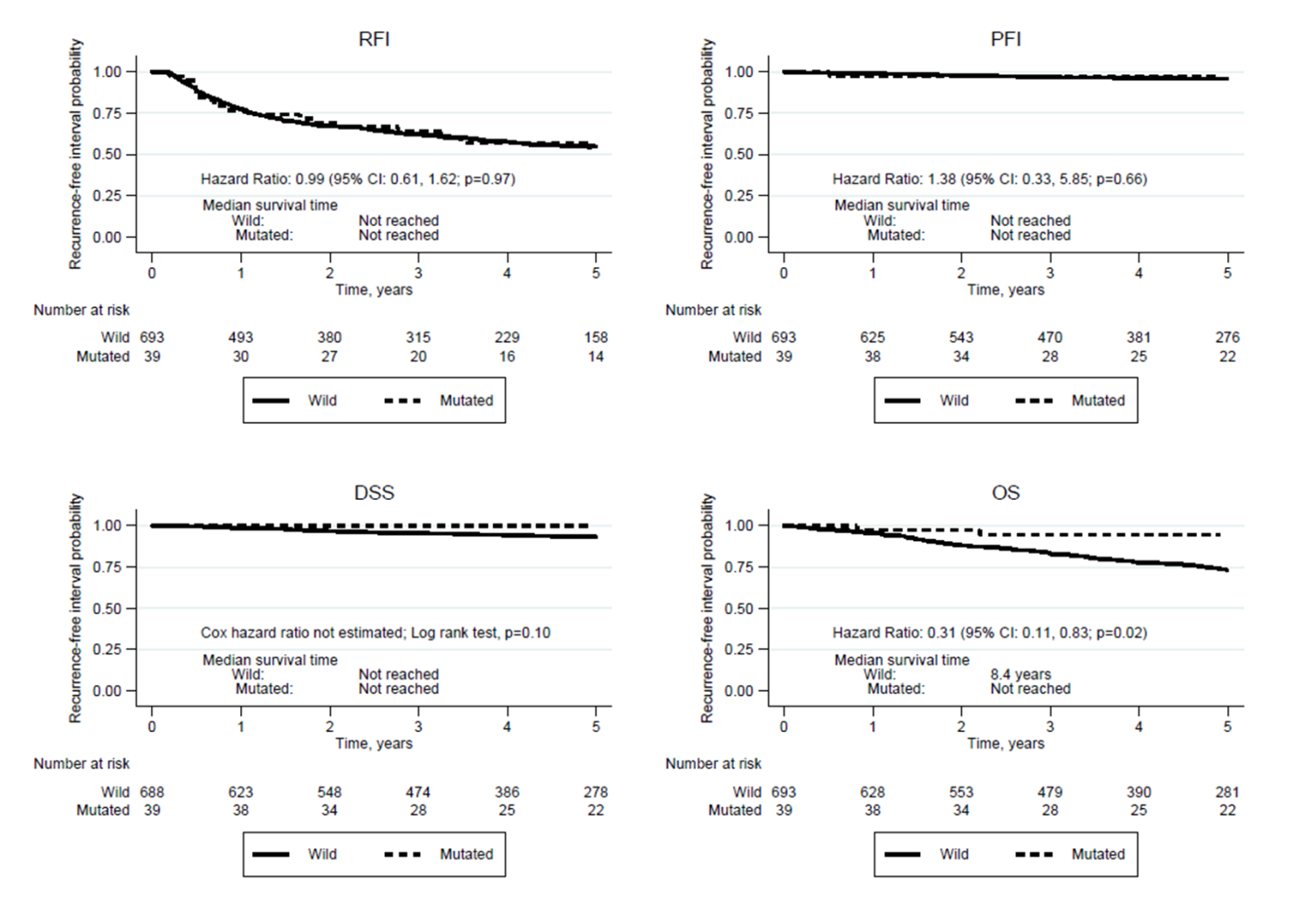


**Figure 10: Kaplan-Meier curves for PIK3CA in the NMIBC population**


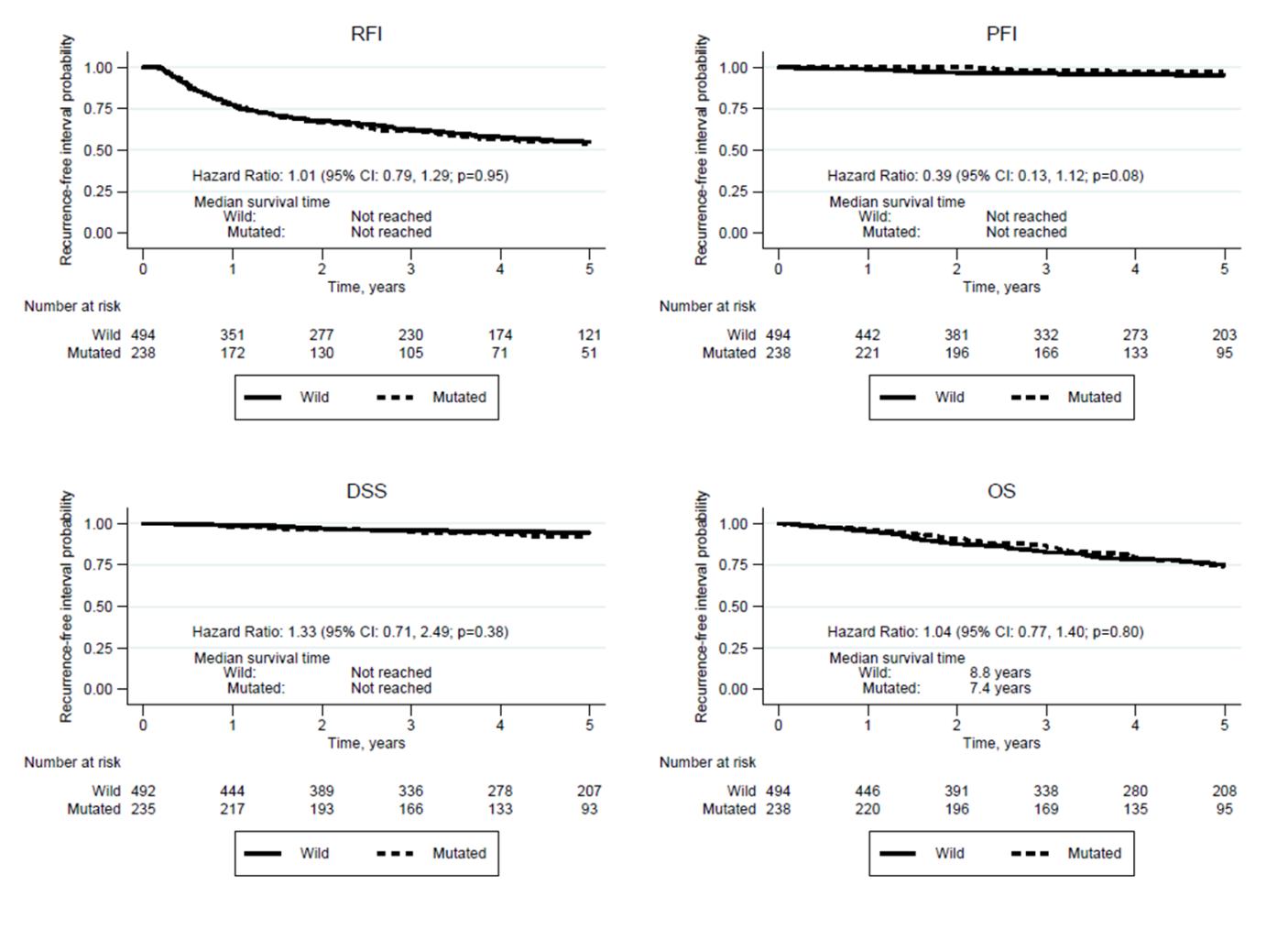


**Figure 11: Kaplan-Meier curves for RAS in the NMIBC population**


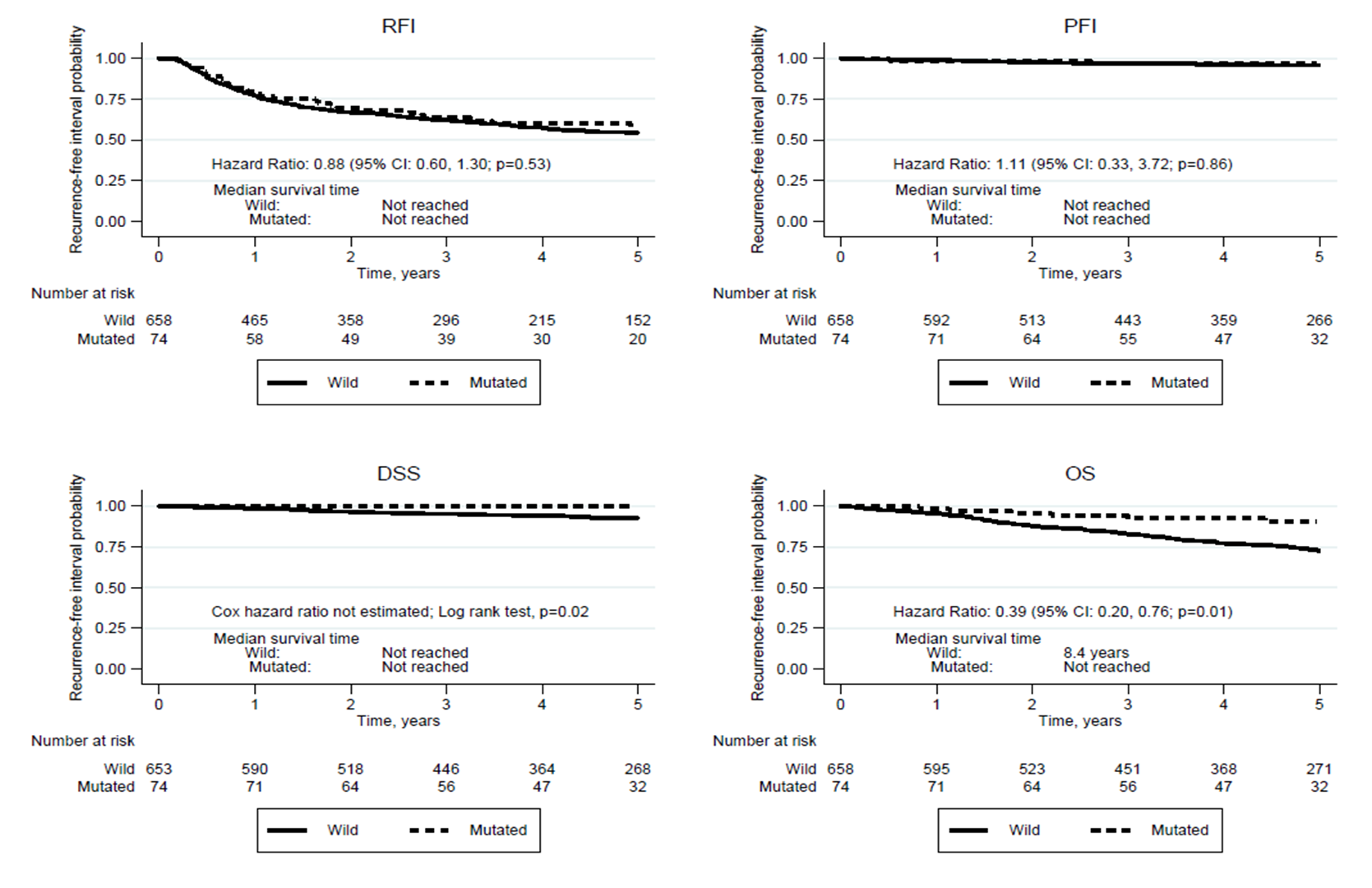


**Figure 12: Kaplan-Meier curves for RHOB in the NMIBC population**


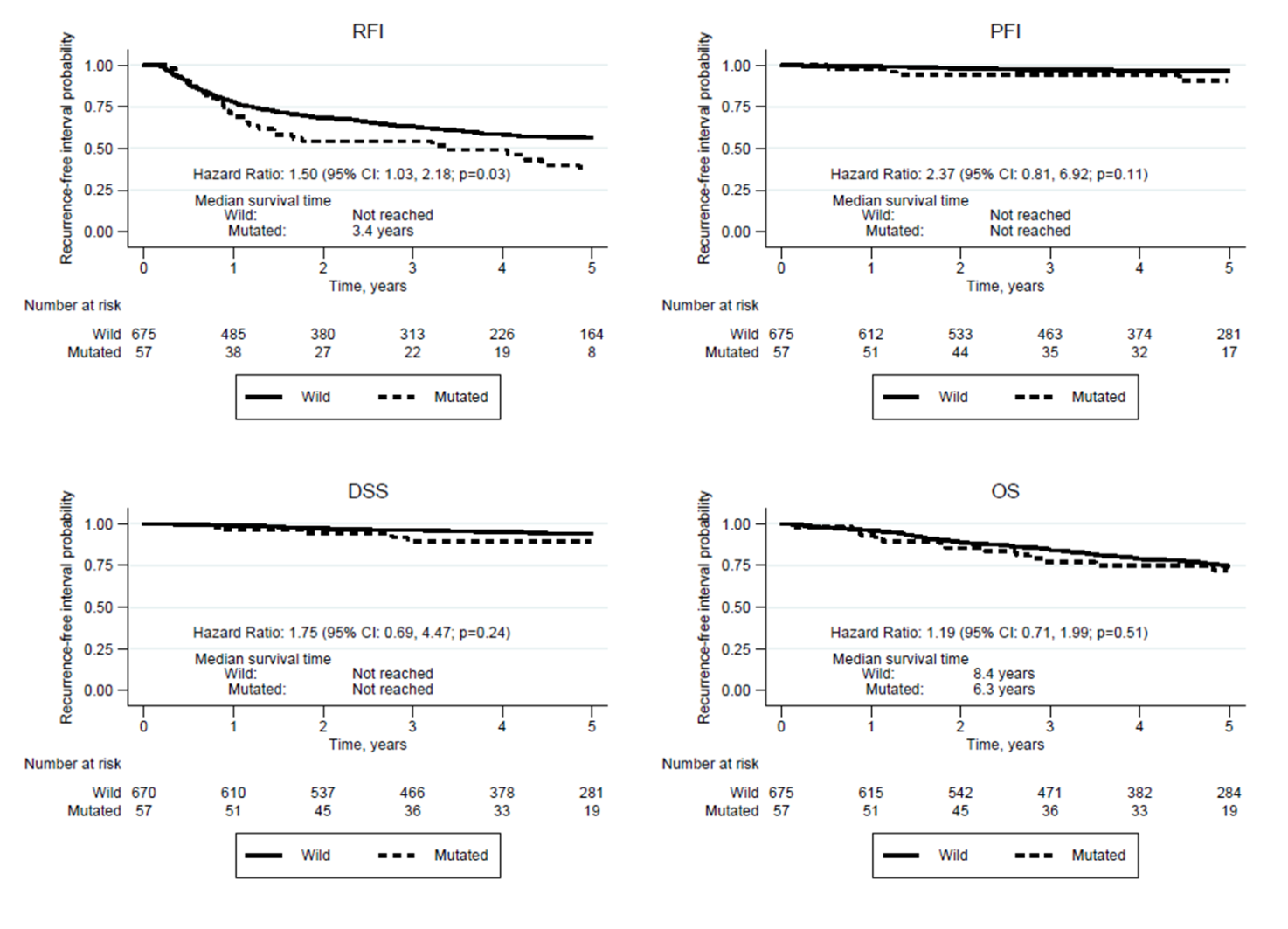


**Figure 13: Kaplan-Meier curves for RXRA in the NMIBC population**


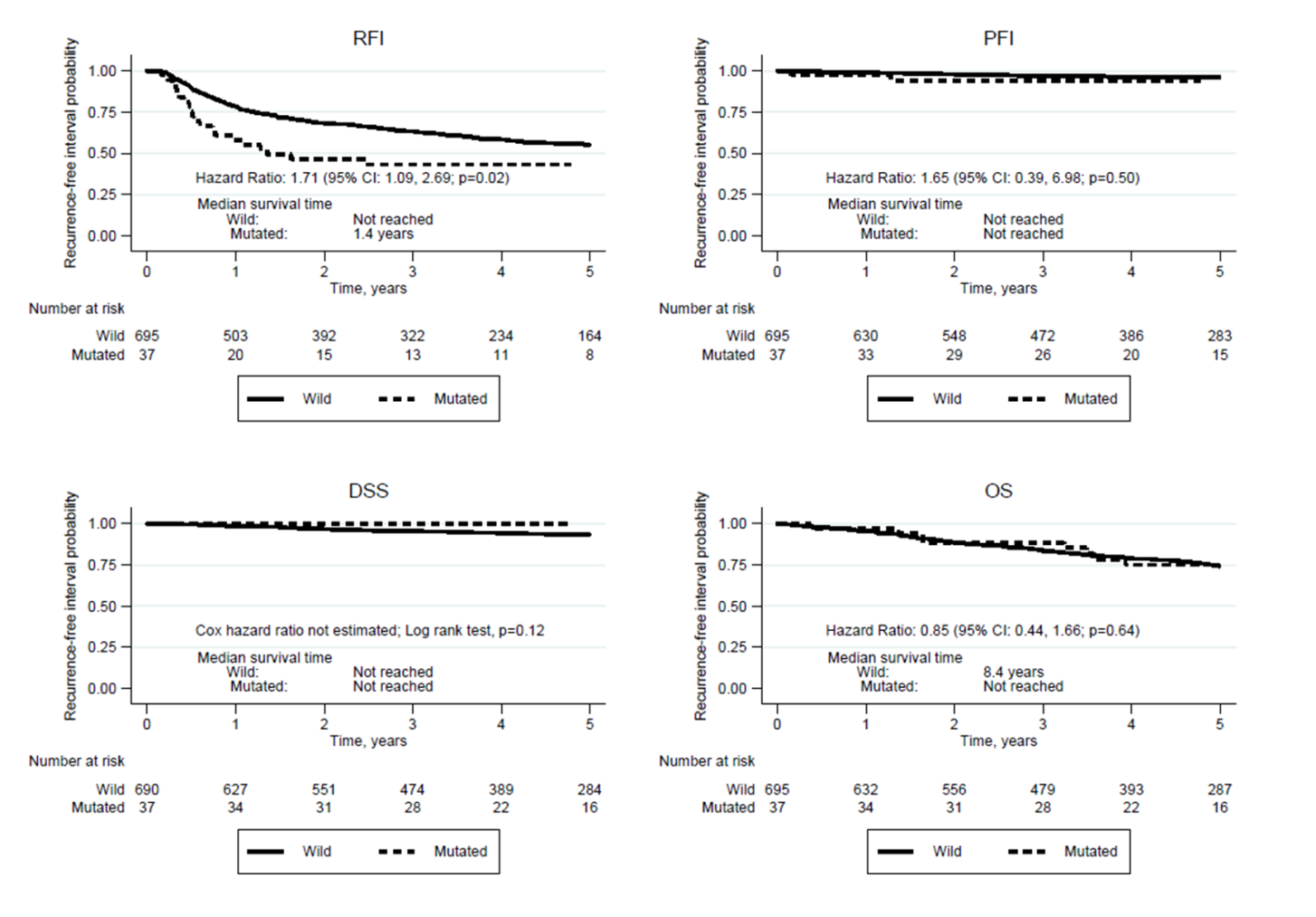


**Figure 14: Kaplan-Meier curves for TERT in the NMIBC population**


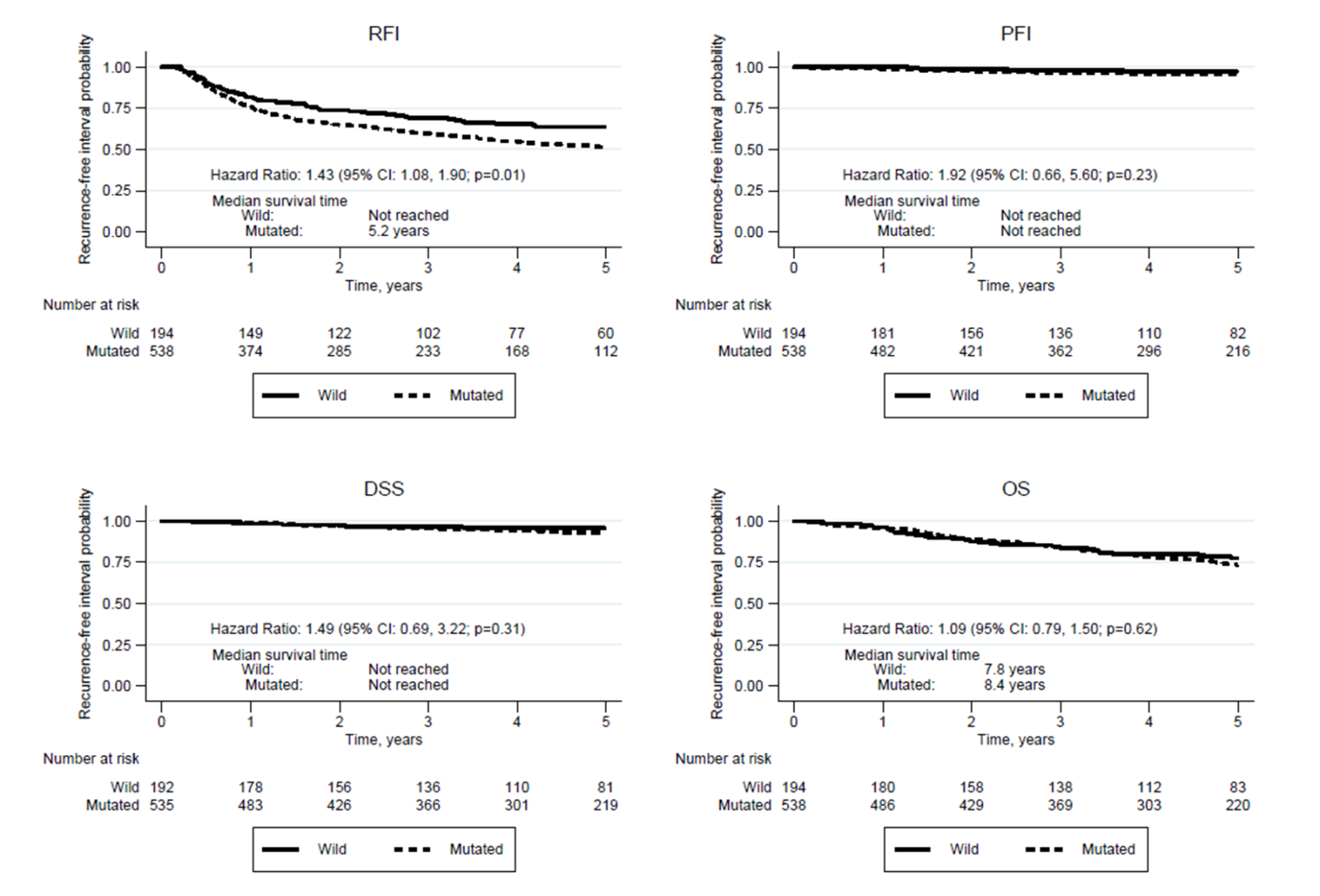


**Figure 15: Kaplan-Meier curves for TP53 in the NMIBC population**


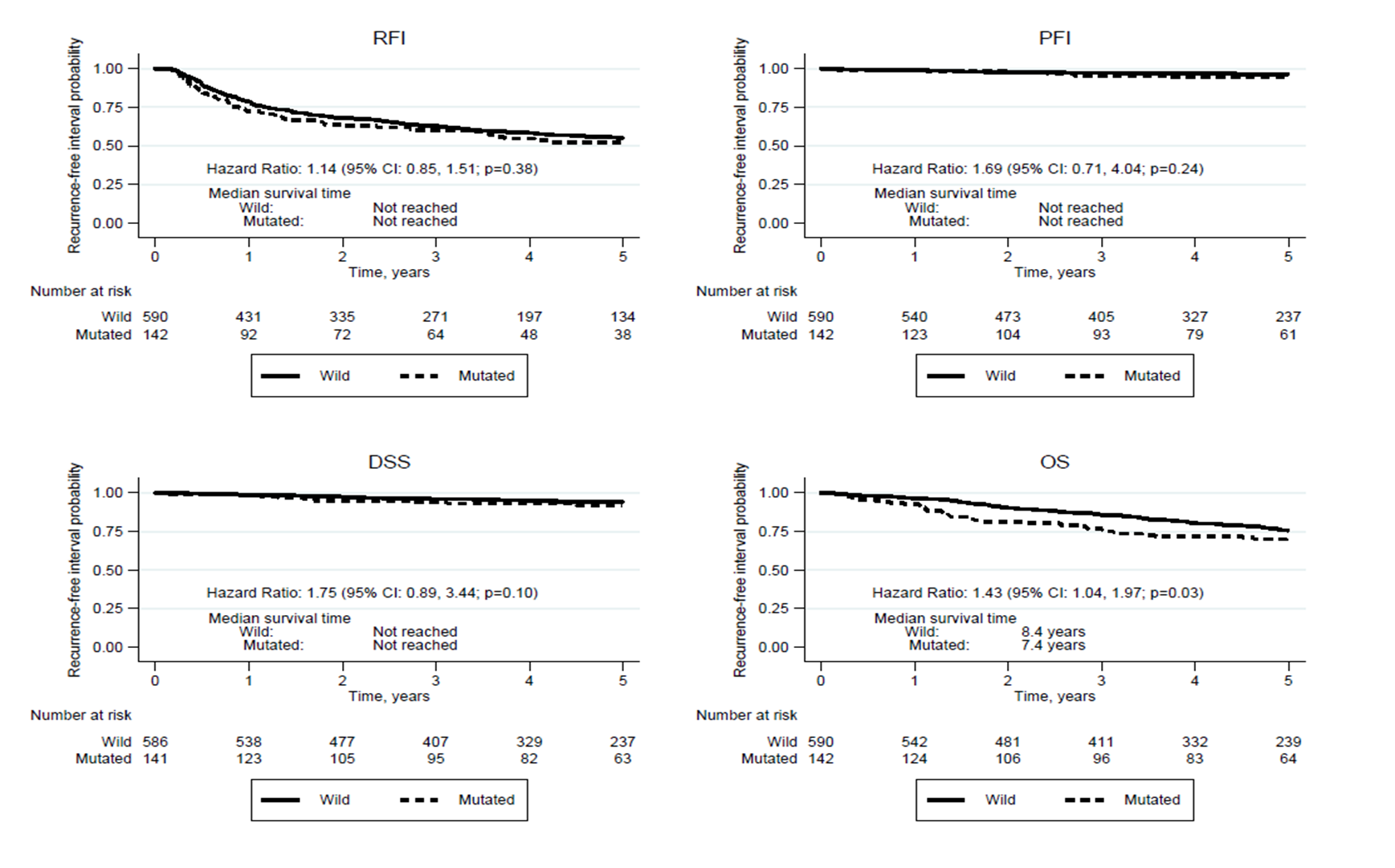


Please note that each row of Table 8 corresponds to a diﬀerent model.

**Table 8: Hazard ratios for the unadjusted and adjusted Cox models for the NMIBC population**

|  | **Mutation** | **Unadjusted model results**  gene incl. as only covariate | | |  |  | **Adjusted model results**  accounts for the sex & risk group* | | |
| --- | --- | --- | --- | --- | --- | --- | --- | --- | --- |
| RFI | ERBB2 | 0.73 | (95% CI: 0.44, 1.20); | p=0.21 |  |  | 0.76 | (95% CI: 0.46, 1.27); | p=0.29 |
|  | ERCC2 | 0.83 | (95% CI: 0.59, 1.16); | p=0.27 |  |  | 0.85 | (95% CI: 0.61, 1.20); | p=0.36 |
|  | FGFR3 | 1.14 | (95% CI: 0.90, 1.44); | p=0.28 |  |  | 1.09 | (95% CI: 0.85, 1.40); | p=0.51 |
|  | HRAS | 0.99 | (95% CI: 0.61, 1.62); | p=0.97 |  |  | 1.07 | (95% CI: 0.65, 1.75); | p=0.79 |
|  | PIK3CA | 1.01 | (95% CI: 0.79, 1.29); | p=0.95 |  |  | 0.99 | (95% CI: 0.77, 1.27); | p=0.94 |
|  | RAS | 0.88 | (95% CI: 0.60, 1.30); | p=0.53 |  |  | 0.96 | (95% CI: 0.65, 1.40); | p=0.82 |
|  | RHOB | 1.50 | (95% CI: 1.03, 2.18); | p=0.03 |  |  | 1.52 | (95% CI: 1.04, 2.20); | p=0.03 |
|  | RXRA | 1.71 | (95% CI: 1.09, 2.69); | p=0.02 |  |  | 1.59 | (95% CI: 1.01, 2.51); | p=0.05 |
|  | TERT | 1.43 | (95% CI: 1.08. 1.90); | p=0.01 |  |  | 1.41 | (95% CI: 1.06. 1.87); | p=0.02 |
|  | TP53 | 1.14 | (95% CI: 0.85, 1.51); | p=0.38 |  |  | 1.19 | (95% CI: 0.88, 1.60); | p=0.27 |
| PFI | ERBB2 | 1.20 | (95% CI: 0.28, 5.08); | p=0.81 |  | Insufficient events to fit model | | | |
|  | ERCC2 | 0.44 | (95% CI: 0.10, 1.85); | p=0.26 |  |  |  |  |  |
|  | FGFR3 | 0.25 | (95% CI: 0.10, 0.62); | p<0.01 |  |  |  |  |  |
|  | HRAS | 1.38 | (95% CI: 0.33, 5.85); | p=0.66 |  |  |  |  |  |
|  | PIK3CA | 0.39 | (95% CI: 0.13, 1.12); | p=0.08 |  |  |  |  |  |
|  | RAS | 1.11 | (95% CI: 0.33, 3.72); | p=0.86 |  |  |  |  |  |
|  | RHOB | 2.37 | (95% CI: 0.81, 6.92); | p=0.11 |  |  |  |  |  |
|  | RXRA | 1.65 | (95% CI: 0.39, 6.98); | p=0.50 |  |  |  |  |  |
|  | TERT | 1.92 | (95% CI: 0.66, 5.60); | p=0.23 |  |  |  |  |  |
|  | TP53 | 1.69 | (95% CI: 0.71, 4.04); | p=0.24 |  |  |  |  |  |
| DSS | ERBB2 | 1.08 | (95% CI: 0.33, 3.49); | p=0.90 |  | Insufficient events to fit model | | | |
|  | ERCC2 | 0.53 | (95% CI: 0.19, 1.48); | p=0.22 |  |  |  |  |  |
|  | FGFR3 | 0.78 | (95% CI: 0.42, 1.45); | p=0.44 |  |  |  |  |  |
|  | HRAS | No hazard ratio estimated*^a^* | | p=0.10 |  |  |  |  |  |
|  | PIK3CA | 1.33 | (95% CI: 0.71, 2.49); | p=0.38 |  |  |  |  |  |
|  | RAS | No hazard ratio estimated*^a^* | | p=0.02 |  |  |  |  |  |
|  | RHOB | 1.75 | (95% CI: 0.69, 4.47); | p=0.24 |  |  |  |  |  |
|  | RXRA | No hazard ratio estimated*^a^* | | p=0.12 |  |  |  |  |  |
|  | TERT | 1.49 | (95% CI: 0.69, 3.22); | p=0.31 |  |  |  |  |  |
|  | TP53 | 1.75 | (95% CI: 0.89, 3.44); | p=0.10 |  |  |  |  |  |
| OS | ERBB2 | 1.52 | (95% CI: 0.94, 2.44); | p=0.08 |  |  | 1.34 | (95% CI: 0.82, 2.19); | p=0.25 |
|  | ERCC2 | 0.91 | (95% CI: 0.62, 1.34); | p=0.63 |  |  | 0.86 | (95% CI: 0.58, 1.27); | p=0.45 |
|  | FGFR3 | 0.88 | (95% CI: 0.66, 1.16); | p=0.35 |  |  | 0.99 | (95% CI: 0.73, 1.34); | p=0.96 |
|  | HRAS | 0.31 | (95% CI: 0.11, 0.83); | p=0.02 |  |  | 0.34 | (95% CI: 0.13, 0.92); | p=0.03 |
|  | PIK3CA | 1.04 | (95% CI: 0.77, 1.40); | p=0.80 |  |  | 1.15 | (95% CI: 0.85, 1.56); | p=0.38 |
|  | RAS | 0.39 | (95% CI: 0.20, 0.76); | p<0.01 |  |  | 0.40 | (95% CI: 0.21, 0.79); | p<0.01 |
|  | RHOB | 1.19 | (95% CI: 0.71, 1.99); | p=0.51 |  |  | 1.21 | (95% CI: 0.72, 2.03); | p=0.46 |
|  | RXRA | 0.85 | (95% CI: 0.44, 1.66); | p=0.64 |  |  | 0.81 | (95% CI: 0.41, 1.58); | p=0.53 |
|  | TERT | 1.09 | (95% CI: 0.79, 1.50); | p=0.62 |  |  | 0.99 | (95% CI: 0.71, 1.37); | p=0.94 |
|  | TP53 | 1.43 | (95% CI: 1.04, 1.97); | p=0.03 |  |  | 1.28 | (95% CI: 0.91, 1.80); | p=0.15 |

*^a^* p-value obtained from log-rank test instead of Cox model

* Risk group is permitted to vary over time.

These analyses only assess the eﬀect of mutation across all NMIBC patients, having accounted for the patient’s risk group, but do not allow the mutation eﬀect to vary for diﬀerent risk groups.

Two mutations (RHOB and TERT) were statistically signiﬁcantly associated with with RFI, after having adjusted for the patient’s sex and risk group. Both of these mutation were associated with an increased risk of recurrence; mutated RHOB increased the risk of recurrence by 52% (HR: 1.52 (95% CI: 1.04, 2.20); p=0.03)) and TERT by 41% (HR: 1.52 (95% CI: 1.04, 2.20); p=0.03)). RXRA was statistically signiﬁcant to 3 decimal places (p = 0.047), and was also associated with an increased risk of recurrence by 59% (HR: 1.59 (95% CI: 1.01, 2.51)).

For PFI and DSS, there were too few events to allow the adjusted models to be ﬁtted.

HRAS and RAS were found to be statistically signiﬁcant for OS in the NMIBC population. These showed a decrease in the risk of death from any cause for patients with a mutation compared to those with wild type. HRAS reduced the all-cause mortality rate by 66% (HR: 0.34 (95% CI: 0.13, 0.92); p=0.03)); RAS reduced the all-cause mortality rate by 60% (HR: 0.40 (95% CI: 0.21, 0.79); p<0.01)).

### 4.5.2 Analyses which account for multiple genes across all NMIBC patients

No models including more than one gene were considered for PFI or DSS, as there were insuﬃcient events to ﬁt the base model with even a single mutation.

Since RHOB, TERT and RXRA all had p-values of less than 0.1 for RFI, there were potentially 4 additional models to be ﬁtted; three pairwise combinations, and one containing all three. The model containing all three was only to be ﬁtted if one of the models including two mutations demonstrated itself superior to models with only a single mutation.

**Table 9: Models for RFI with more than one mutation (adjusting for sex and risk group)**

| **Genes included**  **in model** |  | **Gene** |  | **HR** | **(95% CI)** |  | **Reduction in AIC** |
| --- | --- | --- | --- | --- | --- | --- | --- |
| RHOB & RXRA: |  | RHOB |  | 1.51 | (1.04, 2.20); | p=0.03 | 2.21 |
|  |  | RXRA |  | 1.58 | (1.00, 2.49); | p=0.05 |  |
| RHOB & TERT: |  | RHOB |  | 1.48 | (1.02, 2.15); | p=0.04 | 3.52 |
|  |  | TERT |  | 1.39 | (1.05, 1.85); | p=0.02 |  |
| RXRA & TERT: |  | RXRA |  | 1.50 | (0.95, 2.37); | p=0.08 | 3.20 |
|  |  | TERT |  | 1.38 | (1.04, 1.84); | p=0.03 |  |

Despite both mutations being statistically signiﬁcant in the model including RHOB and TERT, this model cannot be declared as better than the adjusted models containing only one mutation. This is due to the reduction in AIC being small (less than 7), which essentially means that the model with two mutations does not explain enough of the variation to warrant its complexity. Therefore, a model including the three mutations was not ﬁtted.

Since HRAS is essentially a subset of the patients included in RAS, these variables are collinear. As a result, it was not considered appropriate to ﬁt a model for OS containing both of these, and thus no models containing more than one mutation were ﬁtted as HRAS and RAS were the only genes with p< 0.1.

## 4.6 High-risk NMIBC

**Figure 16: Kaplan-Meier curves for PFI in the HR-NMIBC population**


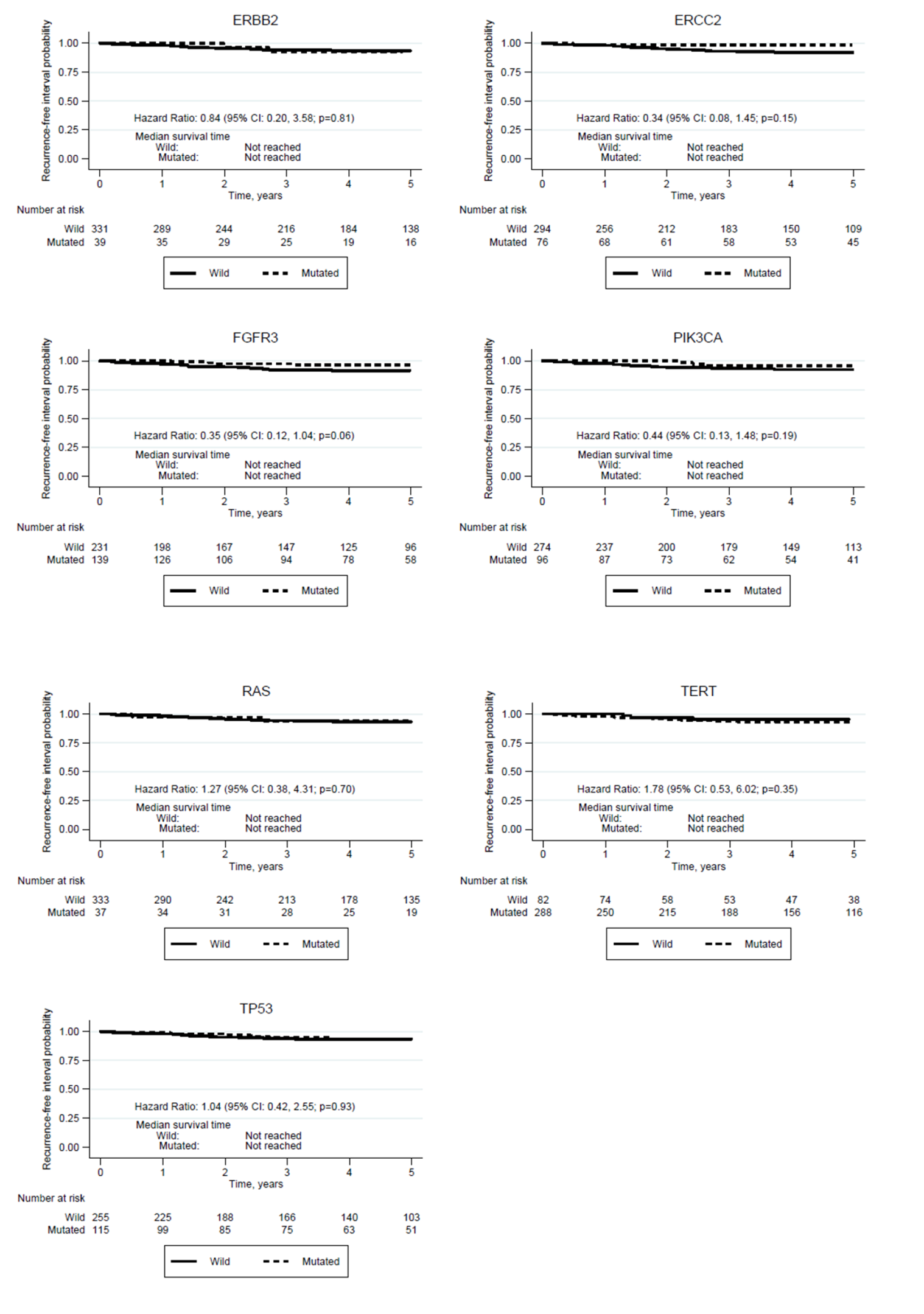


**Figure 17: Kaplan-Meier curves for DSS in the HR-NMIBC population**


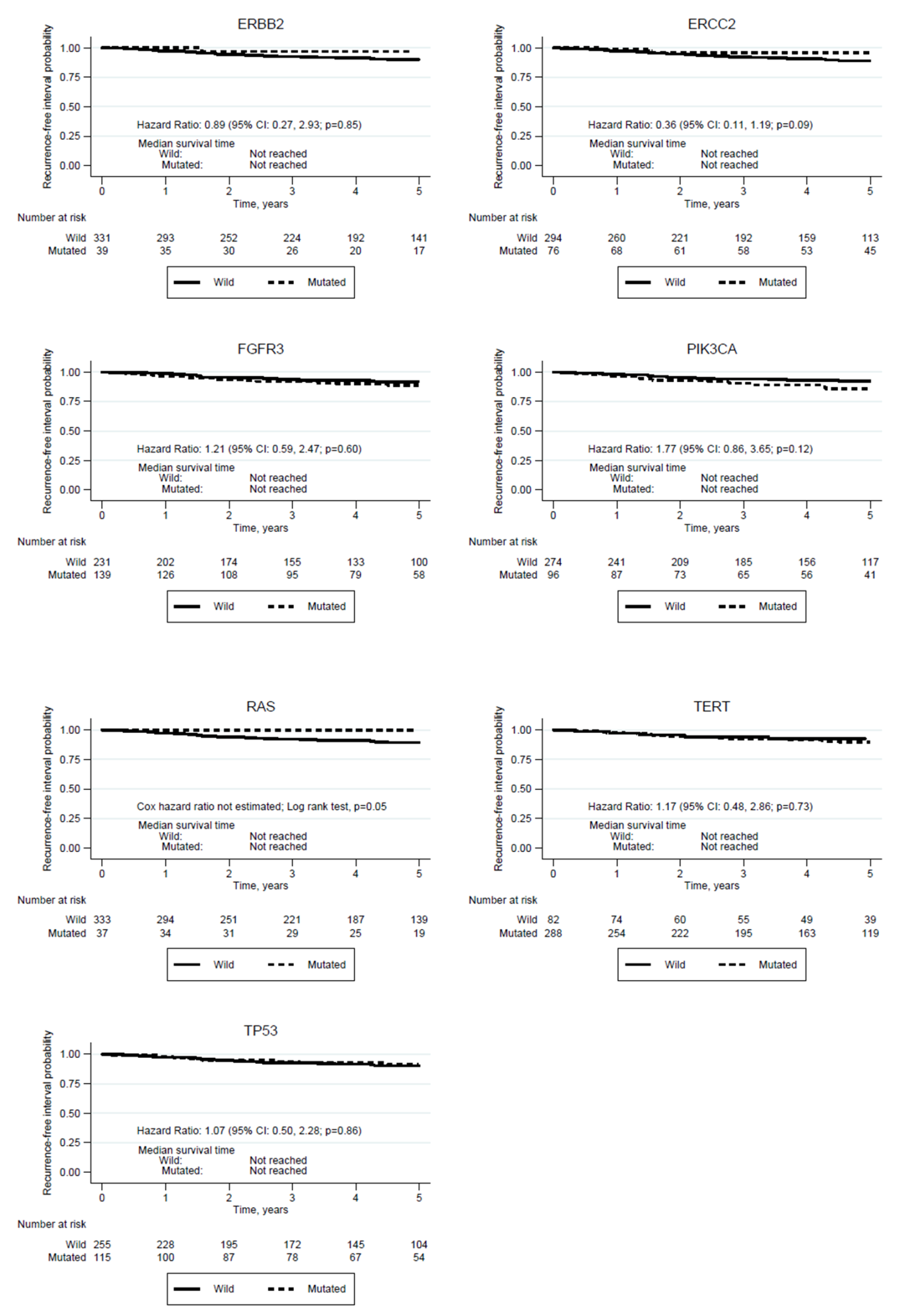


**Table 10: HR-NMIBC models**

|  | **Mutation** | **Unadjusted model results**  gene incl. as only covariate | | |  | **Adjusted model results**  accounts for sex | | |
| --- | --- | --- | --- | --- | --- | --- | --- | --- |
| PFI | CDKN2A | No hazard ratio estimated^a^; | | p = 0.64 |  | Insufficient events for model to calculate HR | | |
|  | ELF3 | 2.34 | (95% CI: 0.69, 7.89); | p = 0.17 |  | 2.27 | (95% CI: 0.67, 7.69); | p = 0.19 |
|  | ERBB2 | 0.84 | (95% CI: 0.20, 3.58); | p = 0.81 |  | 0.89 | (95% CI: 0.21, 3.80); | p = 0.87 |
|  | ERCC2 | 0.34 | (95% CI: 0.08, 1.45); | p = 0.15 |  | 0.33 | (95% CI: 0.08, 1.41); | p = 0.13 |
|  | FGFR3 | 0.35 | (95% CI: 0.12, 1.04); | p = 0.06 |  | 0.35 | (95% CI: 0.12, 1.05); | p = 0.06 |
|  | PIK3CA | 0.44 | (95% CI: 0.13, 1.48); | p = 0.19 |  | 0.46 | (95% CI: 0.14, 1.58); | p = 0.22 |
|  | RAS | 1.27 | (95% CI: 0.38, 4.31); | p = 0.70 |  | 1.23 | (95% CI: 0.36, 4.17); | p = 0.74 |
|  | RHOB | 2.46 | (95% CI: 0.72, 8.34); | p = 0.15 |  | 2.32 | (95% CI: 0.68, 7.90); | p = 0.18 |
|  | RXRA | 1.43 | (95% CI: 0.33, 6.12); | p = 0.63 |  | 1.35 | (95% CI: 0.31, 5.78); | p = 0.69 |
|  | TERT | 1.78 | (95% CI: 0.53, 6.02); | p = 0.35 |  | 1.72 | (95% CI: 0.51, 5.83); | p = 0.38 |
|  | TP53 | 1.04 | (95% CI: 0.42, 2.55); | p = 0.93 |  | 1.04 | (95% CI: 0.43, 2.56); | p = 0.93 |
| DSS | CDKN2A | 3.38 | (95% CI: 0.46, 24.86); | p = 0.23 |  | 3.41 | (95% CI: 0.46, 25.25); | p = 0.23 |
|  | ELF3 | 1.57 | (95% CI: 0.48, 5.18); | p = 0.46 |  | 1.57 | (95% CI: 0.48, 5.18); | p = 0.46 |
|  | ERBB2 | 0.89 | (95% CI: 0.27, 2.93); | p = 0.85 |  | 0.89 | (95% CI: 0.27, 2.95); | p = 0.85 |
|  | ERCC2 | 0.36 | (95% CI: 0.11. 1.19); | p = 0.09 |  | 0.36 | (95% CI: 0.11, 1.18); | p = 0.09 |
|  | FGFR3 | 1.21 | (95% CI: 0.59, 2.47); | p = 0.60 |  | 1.21 | (95% CI: 0.59, 2.47); | p = 0.60 |
|  | PIK3CA | 1.77 | (95% CI: 0.86, 3.65); | p = 0.12 |  | 1.80 | (95% CI: 0.87, 3.73); | p = 0.11 |
|  | RAS | No hazard ratio estimated^a^; | | p = 0.05 |  | Insufficient events for model to calculate HR | | |
|  | RHOB | 2.26 | (95% CI: 0.79, 6.49); | p = 0.13 |  | 2.27 | (95% CI: 0.79, 6.56); | p = 0.13 |
|  | RXRA | No hazard ratio estimated^a^; | | p = 0.12 |  | Insufficient events for model to calculate HR | | |
|  | TERT | 1.17 | (95% CI: 0.48, 2.86); | p = 0.73 |  | 1.17 | (95% CI: 0.48, 2.86); | p = 0.73 |
|  | TP53 | 1.07 | (95% CI: 0.50, 2.28); | p = 0.86 |  | 1.07 | (95% CI: 0.50, 2.28); | p = 0.86 |

*^a^* p-value obtained from log-rank test instead of Cox model

None of the mutations examined were statistically signiﬁcantly associated with either PFI or DSS. However, FGFR3 was close to the cut-oﬀ point (p=0.06) for PFI.
