# Supplementary material for: Targeted deep sequencing of urothelial bladder cancers and associated urinary DNA: a 23‐gene panel with utility for non‐invasive diagnosis and risk stratification
Source: BJU Int. 2019 Jun 19;124(3):532–44. doi: 10.1111/bju.14808 (PMC6772022; doi:10.1111/bju.14808)
Supplement: Supplementary file 4 — Appendix S2. REMARK criteria. [file BJU-124-532-s004.docx]

| **REMARK criteria** | **Answer** | **Notes** |
| --- | --- | --- |
| 1a. Is the marker examined stated | Yes |  |
| 1b. Study objectives stated? | Yes |  |
| 1c. Pre-specified hypothesis stated? | Yes |  |
| 2a. Are patient eligibility characteristics described | Yes | Readers directed to ref. 17 |
| 2b. Source of patients described – intervention? | Yes |  |
| 2c. Source of patients described - control? | N/A |  |
| 2d. Are exclusion criteria stated | Yes | Readers directed to ref. 17 |
| 3a. Treatments described? | Yes |  |
| 3b. How chosen – randomised, rule based, clinician choice? | Yes | Consecutive patients consenting to enrolment in a cohort study (BCPP, ref. 17) |
| 4a. Biological material used - intervention | Yes |  |
| 4b. Biological material used - Control | N/A |  |
| 4c. Preservation/storage described? | Yes |  |
| 5a. Assay methods described? | Yes |  |
| 5b. Assays performed blind to outcome? | Yes |  |
| 6a. Retrospective sampling? | No |  |
| 6b. Prospective sampling? | Yes |  |
| 6c. Recruitment methods consecutive? | Yes | Consecutive patients consenting to enrolment in a cohort study (BCPP, ref. 17) |
| 6d. Recruitment methods random? | No |  |
| 6e. Matched controls? | N/A |  |
| 6f. Study dates reported? | Yes |  |
| 6g. Follow up times reported? | Yes | Supplementary data |
| 7a. All clinical endpoints defined? | Yes | Supplementary data |
| 8a. Candidate variables initially examined or considered for inclusion in models described | Yes | Supplementary data |
| 9a. Sample size given? | Yes |  |
| 10a. Stats methods described? | Yes | Manuscript & Supplementary data |
| 10b. Model building/assumptions described? | Yes | Manuscript & Supplementary data |
| 10c. Missing data handling described? | Yes | Supplementary data |
| 11a. Marker values described? | Yes |  |
| 11b. Cut off points reported? | Yes |  |
| 12a. Flow of patients through the study reported? | Yes |  |
| 12b. Number of dropouts and reasons reported? | Yes | Supplementary data |
| 12c. Subgroup analysis? | Yes | NMIBC & EAU risk groups, MIBC |
| 13a. Demographic characteristics reported? | Yes |  |
| 13b. Missing values reported? | Yes | Supplementary data |
| 14a. Show the relation of the marker to standard prognostic variables? | Yes | Manuscript & Supplementary data |
| 15a. Present univariable analyses showing the relation between the marker and outcome, with the estimated effect (eg, hazard ratio and survival probability). | Yes | Supplementary data |
| 16a. For key multivariable analyses, is the estimated effects reported - e.g. hazard ratio and confidence intervals for the marker | Yes |  |
| 16b. For final model are all variables reported | Yes | Supplementary data |
| 17a. Among reported results, provide estimated effects with confidence intervals from an analysis in which the marker and standard prognostic variable are included, regardless of their statistical significance | Yes | Supplementary data |
| 18a. Are results from further investigations, such as checking assumptions, sensitivity analyses, and internal validation reported? | Yes | Supplementary data |
| 19a. Are results interpreted in relation to the pre-specified hypotheses and other relevant studies. | Yes |  |
| 19b. Are study limitations discussed? | Yes |  |
| 20a. Discuss implications for future research | Yes |  |
| 20b. and clinical value. | Yes |  |
