# Supplementary material for: Targeted deep sequencing of urothelial bladder cancers and associated urinary DNA: a 23‐gene panel with utility for non‐invasive diagnosis and risk stratification
Source: BJU Int. 2019 Jun 19;124(3):532–44. doi: 10.1111/bju.14808 (PMC6772022; doi:10.1111/bju.14808)
Supplement: Supplementary file 5 — Supplementary Material [file BJU-124-532-s005.docx]

*BJU-2019-0383* **TARGETED DEEP SEQUENCING OF UROTHELIAL BLADDER CANCERS AND ASSOCIATED URINARY DNA: A 23-GENE PANEL WITH UTILITY FOR NON-INVASIVE DIAGNOSIS AND RISK STRATIFICATION** D.G. Ward et al.

We thank the Reviewers for their insightful and positive comments on the manuscript, and to be given the opportunity to improve the manuscript. We have endeavoured to address these comments, the specific details of which are in the table below and highlighted in the manuscript.

| **Reviewer Comment** | **Authors’ Response** | **Manuscript lines amended** |
| --- | --- | --- |
| This is highly important work which provides extensive justification and background for a novel genomics platform in bladder cancer. I have few critiques of the work; the authors provide copious supplementary figures but frankly they are all quite useful. | We thank the Reviewer for their positive comments. |  |
| Ward et al have performed a detailed interrogation of urine and tissue specimens derived from patients with bladder cancer. Certain figures in the Supplementary Appendix (e.g., 3.1.1) are incredibly valuable and should be moved to the main study text. | We have now moved the following figures from the Supplementary section into the main text:   - Supplementary Figure S1 becomes **new Figure 1**; - Supplementary Methods & Data Figure 2 (section 3.1.1) becomes **new Figure 2**; and - Supplementary Figure S2 becomes **new Figure 5**.   We have added text to the manuscript where appropriate to introduce these new figures. | Lines 102, 148-151 & 175, respectively. |
| The rates of tissue alterations should be graphically juxtaposed against finding in urine; my speculation is that rate of tissue alterations in certain genes are much higher than detected in urine DNA (in particularly, for FGFR3 and ERBB2, which likely have the most therapeutic relevance at present). | We have added **new Figure 7** which juxtaposes the tissue alterations with those found in both urinary cpDNA and cfDNA.  As the Reviewer speculates, for most alterations, tissue DNA outperforms urine DNA, and we have mentioned this in the Discussion. | Lines 188-190.  Lines 260-264. |
